# Failure of efficient cardiac proteostatic adaptations to chronic cAMP-stress is associated with accelerated heart aging

**DOI:** 10.1007/s11357-025-01851-y

**Published:** 2025-09-01

**Authors:** Maria Grazia Perino, Miguel Calvo-Rubio Barrera, Daniel R. Riordon, Giulio Agnetti, Alexander Maltsev, Admira Parveen, Christopher H. Morrell, Ismayil Ahmet, Khalid Chakir, Yelena S. Tarasova, Jia-Hua Qu, Kirill V. Tarasov, Alexey E. Lyashkov, Yevgeniya O. Lukyanenko, Hikmet Kadioglu, Mark Ranek, Rafael De Cabo, Edward G. Lakatta

**Affiliations:** 1 Laboratory of Cardiovascular Science, Intramural Research Program, National Institute On Aging, National Institutes of Health, 251 Bayview Blvd, Baltimore, MD 21224 USA; 2 Translational Gerontology Branch, Intramural Research Program, National Institute On Aging, National Institutes of Health, Baltimore, MD USA; 3 Division of Cardiology, Department of Medicine, Johns Hopkins University School of Medicine, Baltimore, MD USA; 4 Department of Biochemistry and Molecular Biology, Johns Hopkins University School of Public Health, Baltimore, MD USA; 5 Jude Children’s Research Hospital, Memphis, TN USA; 6 Dipartimento Di Scienze Biomediche E Neuromotorie (DIBINEM), Università Di Bologna, Bologna, Italy

**Keywords:** Aging, Dysregulated proteostasis, Protein quality control

## Abstract

Dysregulated proteostasis is a hallmark of aging. We investigated how efficiently proteostatic adaptations to chronic cardiac cyclic-adenosine-monophosphate (cAMP)-dependent stress change with aging in mice harboring marked cardiac-specific over-expression of adenylyl cyclase VIII (TG^AC8^). We assessed protein quality control mechanisms (PQC) (ubiquitin proteasome system, autophagic flux via macroautophagy, and mitophagy) in left ventricles of TG^AC8^ and wild-type littermates (WT) at 3–4 and 17–21 months of age. At 3–4 months, TG^AC8^ exhibited markers of increased autophagic flux (*microtubule-associated protein 1A/1B light chain 3B* (LC3), p62, and their phospho-forms) and enhanced canonical mitophagy signaling (PARKIN, p62^S405^ and p62^S349^ receptors), confirming a more efficient proteostasis, vs WT. In aged TG^AC8^, however, the PQC mechanisms were overwhelmed by proteotoxic stress, manifested in insufficient proteasome activity, slower autophagic flux, and increased mitochondrial dysfunction (network fragmentation). The accumulation of protein aggregates (increased ratio of insoluble/soluble protein fractions), of lipofuscin bodies and of desmin cardiac preamyloid oligomers, and of LC3^+^- and p62^+^-inclusions of aberrant sizes was increased in aged TG^AC8^ compared to young TG^AC8^. Thus, while increased proteostatic mechanisms maintain cardiac health in TG^AC8^ in youth (3–4 months), long-term exposure to sustained activation of the AC/cAMP/PKA/Ca^2+^ signaling axis results in severe proteostasis insufficiency in aged TG^AC8^, leading to cardiomyopathy and accelerated cardiac aging.

**Graphical Abstract:**

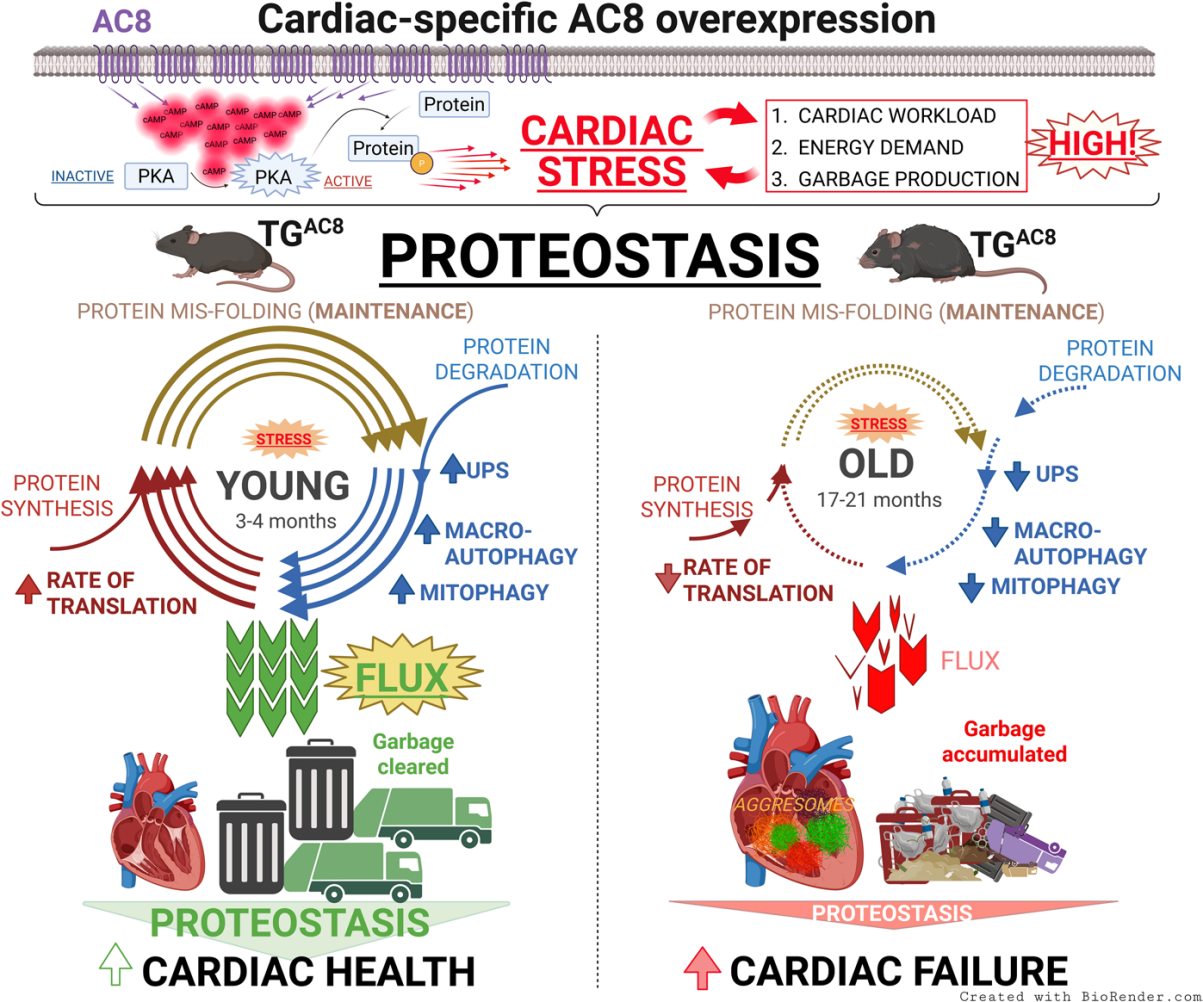

**Supplementary Information:**

The online version contains supplementary material available at 10.1007/s11357-025-01851-y.

## Introduction

Cardiac protein quality control (PQC) is required for the maintenance of cardiac health and function. Pathogenic aspects of protein misfolding and aggregation, collectively referred to as *proteotoxicity*, accompany advancing age and can ultimately lead to heart dysfunction [1] and eventual cardiac failure [2]. Many protein aggregate–based diseases in the heart have been well characterized [3]. Plaque deposition (amyloidosis) or accumulation of soluble, oligomeric aggregates, and, more recently, desmin disorganization and the increase in cardiac desmin-preamyloid oligomers (PAOs) [4] have also emerged as common hallmarks of aging and acquired heart failure (HF) [5, 6].

Many factors, including an imbalance of protein synthesis and degradation, exhaustion of proteostatic machinery, and downregulation of clearing mechanisms, e.g., the ubiquitin proteasome system (UPS) [7] and autophagy [8], can lead to collapse of proteostasis. Impaired autophagy and mitophagy that accompany advancing age due to reduced lysosomal [9] and mitochondrial fitness resulting from increased oxidative stress [10] have been linked to the accumulation of electron-dense aggregates, generally referred to as lipofuscin (LF). As age advances, accumulation of LF occurs as PQC mechanisms become insufficient and begin to collapse [11]. The linear increase of LF as age advances is inversely correlated with longevity [12].

Our recent study demonstrated that chronic severe cardiac stress imposed by over-expression of adenylyl cyclase VIII (AC8) in mice (TG^AC8^) induced a concentric pattern of adaptive signaling circuitry at a young age (3–4 months) [13]. This circuitry included impressive upregulation of protein synthesis and degradation mechanisms (proteasome and autophagy) that protect against cardiac stress, maintaining the remarkably high performance of the heart for up to about 1 year of age [14, 15]. However, signs of HF and cardiomyopathy begin to emerge in TG^AC8^ as age advances [16]. Here, we tested the hypothesis that the adaptive proteostatic mechanisms in response to severe sustained cAMP-dependent chronic cardiac stress become *more impaired* (overwhelmed) in TG^AC8^ than in WT in advanced age.

## Methods

An expanded “Methods” section is available in the supplemental materials.

### Experimental animals

Breeding pair of mice harboring human AC8 under the murine α-myosin heavy chain (α-MHC) promoter (TG^AC8^), and WT (background strain C57BL/6 from Jackson Labs, Stock # 000664), were a gift from Nicole Defer/Jacques Hanoune, Unite de Recherches, INSERM U-99, Hôpital Henri Mondor, F-94010 Créteil, France [14]. HAC8^+^ mice were crossed with C57Bl6 mice. Our study exclusively examined male mice. It is unknown whether the findings are relevant for female mice. All assays were performed in 3–4-month-old (young) and 17–21-month-old (old) TG^AC8^ and compared to age-matched WT. Mice were sacrificed using ketamine/xylazine mixture, by intraperitoneal (IP) administration.

### Heart and cardiac tissue isolation

The heart was quickly removed and placed into cold PBS solution. The left ventricle (LV) free wall, without the septum, was identified anatomically under a dissecting microscope, excised, snap-frozen in liquid nitrogen, and stored at − 80 °C for further analyses.

### Protein extraction and WB

Flash-frozen tissue was homogenized as previously described [13]. Briefly, snap-frozen LVs were homogenized and lysed in ice-cold RIPA buffer supplemented with Halt protease and Halt phosphatase inhibitor cocktails (all Thermo-Fisher Scientific), and 1 mM phenylmethyl sulfonyl fluoride (Sigma-Aldrich), using a Precellys homogenizer with a tissue homogenization kit CKMix (Bertin Instruments) at 4 °C. Lysates were then centrifuged at 10,000 g for 10 min at 4 °C to separate insoluble material. Nuclear and cytosolic lysates were prepared from snap-frozen LV using a subcellular protein fractionation kit for tissues (Thermo-Fisher Scientific) as per the manufacturer’s instructions. The protein concentration of samples (soluble fractions) was determined using the Bicinchoninic Acid (BCA) Assay (Thermo-Fisher Scientific), whereas for insoluble fractions (pellets), the EZQ Protein Quantitation Kit (Molecular Probes) was used. Samples were denatured in 4X Laemmli sample buffer (BioRad Laboratories) containing 355 mM 2-mercaptoethanol at 95 °C for 5 min, DTT or NuPage Reducing Agent 10X (Invitrogen) at 70 °C for 10 min, and resolved on 4–20% Criterion TGX Stain Free gels (BioRad Laboratories) by SDS/PAGE. For HSP60, lysates were denatured in XT sample buffer containing 1X XT reducing reagent (BioRad Laboratories) at 95 °C for 5 min and proteins resolved on a 12% Criterion™ XT Bis–Tris gel using XT MES running buffer (BioRad Laboratories). For Stain-Free™ gels, to induce crosslinking of trihalo compound with protein tryptophan residues, gels were exposed to UV transillumination for 2–3 min. Proteins were then transferred to low-fluorescence polyvinylidene-difluoride (LF-PVDF) membranes (BioRad Laboratories), using an electrophoretic transfer cell (Mini Trans-Blot, BioRad Laboratories). Membrane total protein was visualized using an Amersham Imager 600 (AI600) (GE Healthcare Life Sciences) with UV transillumination. Membranes were blocked with either 5% milk/tris-buffered saline with Tween-20 (TBST) or EveryBlot Blocking Buffer (BioRad Laboratories) as appropriate and were then incubated with the primary antibodies indicated in Table 1. Primary antibodies were then detected using horseradish peroxidase (HRP) conjugated antibody (Invitrogen) at 1:10,000, and bands were visualized using Pierce SuperSignal West Pico Plus ECL substrate kits (Thermo-Fisher Scientific). The area of interest was selected and the signal captured (semi-auto mode) using an Amersham Imager 600 (AI600) (GE Healthcare Life Sciences). Total protein for HSP60 blot was visualized by staining membrane with 1X Amido Black solution (Millipore Sigma) according to manufacturer’s protocol. Band density was quantified using ImageQuant TL software (GE Healthcare Life Sciences). Total protein was used as control for protein loading. Membranes were stripped and reprobed several times.

**Table 1:** Primary antibodies used in WB and IHC

Target protein	Company	Cat. No
Primary antibodies used in WB analyses		
ACP2	Proteintech	15236–1-AP
ALIX/PDCD6IP	Proteintech	12422–1-AP
ATG16L1	Abcam	ab187671
ATG4B	Abcam	ab199537
ATG9A	Abcam	ab108338
DRP1	Cell Signaling Technology	8570
FKBP8	ThermoFisher Scientific	PA5-96573
HSP60	Abcam	ab190828
HSP90a	ThermoFisher Scientific	PA3-013
LC3A	Abcam	ab52628
LC3B	Novus Biologicals	NB100-2220
LC3A/B	Cell Signaling Technology	4108
LC3^S12^	Bio Vision	6951
LC3^T50^	Abcam	ab204297
MFN1	Abcam	ab221661
OPA1	ThermoFisher Scientific	MA5-16149
p62/sequestosome 1	Abcam	ab56416
p62^S349^	Cell Signaling Technology	16177
p62^S403^	Cell Signaling Technology	39786
PARKIN	ThermoFisher Scientific	39–0900
Puromycin	Sigma	MABE343
TBP	Cell Signaling Technology	8515
TFEB	ThermoFisher Scientific	PA5-96632
TFEB^S211^	Cell Signaling Technology	37681
Vinculin	Cell Signaling Technology	13901
Primary antibodies used in IHC		
HSP27^S82^	Cell Signaling Technology	9709
LAMP1	DSHB	1D4B
LC3A/B	Cell Signaling Technology	12741
p62/sequestosome 1	Abcam	ab56416
Wheat Germ Agglutinin (WGA)	ThermoFisher Scientific	W21404

### Immunostaining (IHC)

Endogenous puncta were determined in 5-µm-thick paraffin sagittal sections of LV tissue. Following standard procedures, deparaffinization and rehydration were followed by heat antigen-retrieval in a citric acid base solution (H-3300, Vector-Labs). Autofluorescence was quenched with a 10-min incubation with 1 mg/ml sodium borohydride solution in PBS. Sections were blocked in 10% goat serum for 30 min and incubated at 4 °C overnight with the primary antibodies indicated in Table 1. After incubation with corresponding conjugated secondaries and counterstaining with 500 nM 4′,6-diamidino-2-phenylindole (DAPI) for about 1 h at RT, immunolabeled samples were mounted using an antifade gel mounting medium (Vectashield Vibrance; Vector-Labs) and examined with a Zeiss LSM 900 confocal microscope. At least 10 random fields of optical regions (100 µm^2^) of 5 µm thickness per cardiac section (*n* = 3) were collected with a Plan-Apochromat 63x/1.40Oil DIC M27 objective and projected on a single extended projection image to analyze. Puncta counting and areas of vesicles were measured using manual tracking with ImageJ software by a blinded investigator.

### Transmission electron microscopy (TEM)

Samples for TEM were fixed using 2.5% glutaraldehyde in 0.1 M sodium cacodylate buffer, pH = 7. Then, they were post-fixed in 1% osmium tetroxide for 1 h at 4 °C in the same buffer, dehydrated, and then embedded in Embed 812 resin (Electron-Microscopy Sciences) through a series of resin resin-propylene oxide gradients to pure resin. Blocks were formed in fresh resin contained in silicon molds, and the resin was polymerized for 24–48 h at 65 °C; blocks were then trimmed and sectioned in an EM UC7 ultramicrotome (Leica Microsystems) to obtain both semi-thick (0.5–1 µm width) and ultrathin (40–60 nm width) sections. Semi-thick sections were mounted on glass slides and stained with 1% toluidine blue in a 1% borax aqueous solution for 2 min. Palade-stained (OsO4) and toluidine blue-stained semi-thick sections were then imaged using a Leica AXIO Imager light microscope with a 63 × oil immersion objective, for quality control and LF-quantification. Ultrathin sections were stained with uranyl acetate and lead citrate and then imaged on a Talos L120C TEM Microscope with a 4 K Ceta CMOS camera, for macroautophagic events quantification. Micrographs at × 2000, × 5000, and × 11,000 magnification were obtained from randomly selected cytoplasmic areas of cardiomyocytes, for illustration purposes and quantitative analysis of macroautophagic events population. Two stereological parameters were determined: (a) the numerical profile density Na (number of figures of interest/µm^2^ of cell-fraction) and (b) the volume density of figures of interest (Vv; i.e., the volume fraction of cardiomyocyte cytoplasm occupied by figures of interest). Volume density was obtained following a point analysis using a simple square lattice test system [17], with superposition of a virtual grid over the micrographs where the user performs a point-counting method. Autophagosomes (*early*-events) were identified as double-membrane vesicles with identifiable cargo and comparable density to the surrounding cytosol. Autolysosomes (*late*-events) and residual bodies were denoted as single membrane vesicles containing non-identifiable cargo of higher density than the surrounding cytosol and fragmented organelles. LF bodies were identified following Palade staining [18] by the presence of densely packed lipids with a dark brown-black color against cardiomyocyte cytosol, which allowed a straightforward segmentation of the image and posterior quantification of the planimetric and stereological parameters. Point counting and areas of vesicles and cytosol were measured using manual tracking with ImageJ software by a blind investigator.

### RT-qPCR

RNA was extracted from LVs with RNeasy Mini Kit (Qiagen) and DNAse on-column digestion. One microgram of total RNA was used for cDNA synthesis with MMLV reverse transcriptase (Promega). RT-qPCR was performed using a QuantStudio 6 Flex Real-Time PCR System (Thermo-Fisher Scientific) with a 384-well platform. The reaction was performed with a FastStart Universal SYBR Green Master Kit with Rox (Roche) using the manufacturer’s recommended conditions; the sizes of amplicons were verified. Each well contained 0.5 µl of cDNA solution and 10 µl of reaction mixture. Each sample was quadruplicated and repeated twice using de novo synthesized cDNA sets. Preliminary reactions were performed to determine the efficiency of amplification. RT-qPCR analysis was performed using the ddCt method. Primers were selected with Primer Express 3.0 software (Applied Biosystems). The primers sequence is indicated in Table 2.

**Table 2:** List of primers used in qPCR

Primers for qPCR		
Target gene	Sequence	Amplicon size
HPRT. F	CTTCCTCCTCAGACCGCTTTT	97 bp
HPRT. R	CATAACCTGGTTCATCATCGCTAA	
YWHAB. F	GCGCTGAATTTCTCAGTCTTTTACT	96 bp
YWHAB. R	CTCAGCAATCGCCTCATCAA	
YWHAE. F	TGAGGCGCCGCCATT	78 bp
YWHAE. R	ACGGATGGAAGCGGATAGC	
YWHAH. F	ATCTGTATTGGCAGCACAGCTATT	77 bp
YWHAH. R	GCCCATGAAGGTTTATCTGAAACT	
YWHAG. F	GCCAAGACCGCCTTCGA	83 bp
YWHAG. R	TGCATGATCAGAGTGGAGTCCTT	
YWHAQ. F	GGCGATGATCGAAAACAAACA	80 bp
YWHAQ. R	TGCATCTCCTTCTTGCTTATATCAA	
YWHAZ. F	AAGGCCTGGAGCACTTGTGA	84 bp
YWHAZ. R	CAAGAGTGTGCACGCAGACA	
Sfn. F	CCGCAGAACCCAGCGTTA	65 bp
Sfn. R	GACTGCGAGGATGGACAGACA	

### AI algorithm for analysis of mitochondrial parameters

Fifteen TEM images were acquired from the LV of each of 40 adult rodents, yielding 600 images in total. All images were downsampled by a factor of 4 to balance spatial detail with processing speed. One representative image per animal was annotated by a marker-guided, high-quality Segment Anything model (HQ-SAM ViT-L) (Ke L et al. arXiv:2306.01567 10.48550/arXiv.2306.01567), producing 40 expert ground-truth masks. These masks trained a nnU-Net semantic segmentation network [19] on 32 images with 8 held out for validation, achieving a mean Dice similarity coefficient of 0.95. Due to occasional segmentation failures or image quality issues, an average of 13.7 images per animal passed all quality controls and were carried forward. Instance segmentation proposals were generated on each image using the SAM automatic mask generator with 24 sampling points per side to ensure uniform coverage. Each proposal was then evaluated by an internal quality estimator that measures alignment with image edges and textures and by a stability score based on consistency across sampling runs. Any proposal scoring below 0.86 on quality or below 0.92 on stability was discarded. Next, a single hierarchical crop pass subdivided each image into overlapping tiles at multiple scales, preserving sampling density to capture fine structures, and eliminated any tile smaller than 10 pixels to avoid spurious fragments. Proposals from both the original image and the semantic-mask-filtered image were merged by assigning each overlapping pixel to the smaller region, consolidating candidate masks while conserving detailed boundaries. Postprocessing extracted the largest connected component for each merged region, filled internal holes, and applied size and shape filters to exclude extremely small or irregularly shaped mitochondria, yielding a refined set of high‑confidence instance masks for downstream analysis. Finally, mitochondria that were touching the border of the TEM image were removed from analysis. To ensure that only mitochondrial pixels contributed to measurements, each set of instance masks was intersected with the binary semantic output. For the 40 validation images, metrics were computed on both automated and manual masks and expressed their agreement as a percent difference (absolute deviation divided by the ground-truth value × 100). To ensure that only mitochondrial pixels contributed to measurements, each set of instance masks was intersected with the binary semantic output. For the 40 validation images, metrics were computed on both automated and manual masks and expressed their agreement as a percent difference (absolute deviation divided by the ground‑truth value × 100). Averaged across all images, the mean percent differences were as follows: − *mean area (µm*^*2*^*): 10.67%, − median area (µm*^*2*^*): 11.10%, − object count: 11.49%, − summed object area (µm*^*2*^*): 2.58%, − mean perimeter (µm): 8.48%, − median perimeter (µm): 7.72%, − mean circularity: 3.72%, − median circularity: 2.45%, − number density Na (objects per cardiomyocyte area): 8.45%, − area fraction Aa (mitochondrial area per cardiomyocyte area): 2.82%*. These low percent differences demonstrate that the combined HQ‑SAM and nnU‑Net workflow closely replicates expert manual delineations. The table of statistics is shown in Supplemental Table 1.

### Echocardiography

Mice underwent echocardiographic (echo) examination (40-MHz transducer; Visual Sonics 3100; Fuji Film Inc) under light anesthesia with isoflurane (2% in oxygen) via nosecone; temperature was maintained at 37 °C using a heating pad. Mice were placed in the supine position; skin hair in the chest area was shaved. Standard electrocardiogram (ECG) electrodes were placed on the limbs, and ECG Lead II was recorded simultaneously with the acquisition of echo images. Each echo examination was completed within 10 min. Parasternal long-axis views of the LV were obtained and recorded to ensure that mitral and aortic valves and the LV apex were visualized. From the parasternal long-axis view of the LV, M-mode tracings of LV were obtained at mid-papillary muscle level. Parasternal short-axis views of the LV were recorded at the mid-papillary muscle level. Endocardial area tracings, using the leading-edge method, were performed in the 2D mode (short-axis and long-axis views) from digital images captured on a cine loop to calculate the end-diastolic and end-systolic LV areas. LV end-diastolic volume (EDV) and end-systolic volume (ESV) were calculated by a Hemisphere Cylinder Model method. Ejection fraction (EF) was derived as EF = 100*(EDV-ESV)/EDV. LV mass (LVM) was calculated from EDV, inter ventricular septal thicknesses (IVS, measured from the LV-M-mode tracing), and LV posterior wall (PW). All measurements were made by a blinded, single investigator and are reported as the average of five consecutive cardiac cycles covering at least one respiration cycle (100 times/min in average). The reproducibility of measurements was assessed by repeated measurement a week apart in randomly selected images; the repeated measure variability was less than 5%.

### Statistics

Statistical analyses were performed in GraphPad Prism 10 and R. ROUT analyses were applied to identify outliers, which were excluded from group comparison analyses. Student *t* test with Welch’s correction was used to compare data between two groups. Two-way ANOVA followed by original FDR method of Benjamini and Hochberg or Fisher’s LSD post-hoc test were used for multiple comparisons [20]. For datasets with matching LC3I and LC3II from the same animal, a repeated-measures approach with three-way mixed ANOVA or mixed-effect analysis for repeated-measures was taken, followed by original FDR method of Benjamini and Hochberg. Statistical significance was assumed at *p* < 0.05.

### Study approval

Mice were housed in a climate-controlled room with 12-h light-cycle and free access to food and water, as previously described [21], in accordance with NIH guidelines. The protocol was approved by the NIH Animal Care and Use Committee (ACUC) (441-LCS-2025).

## Results

### Autophagy is upregulated and autophagic flux is optimized in the young TG^AC8^heart (3–4 months)

To assess basal autophagy in young TG^AC8^ and WT, we performed WB of the established autophagy markers LC3 and p62/sequestosome-1 and assessed their activity via measuring their phosphorylation states. Both LC3A and LC3B isoforms, which localize on different autophagosomes [22] and exhibit distinct expression patterns and functions [23], were assessed using isoform-specific antibodies.

**Fig. 1:**
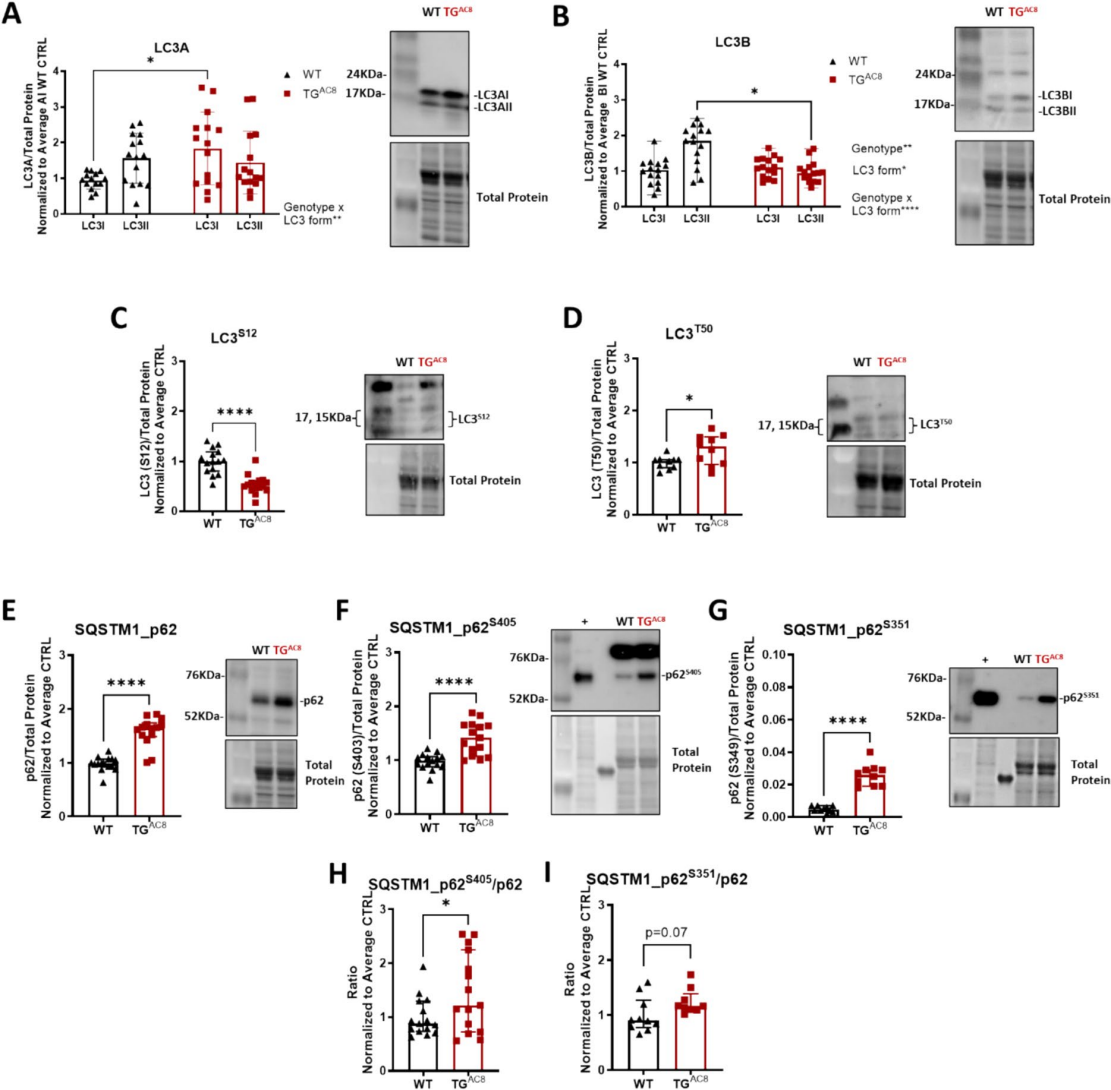
Autophagy is upregulated in TG^AC8^ at 3–4 months of age. Bar graphs and WB of LC3 and p62 and their phosphoforms in young TG^AC8^ and WT (*n* = 15 mice/group). **A** LC3A; **B** LC3B; **C** LC3^S12^; **D** LC3^T50^; **E** p62; **F** p62^S405^; **G** p62^S349^; **H** p62^S405^/p62 ratio; **I** p62^S349^/p62 ratio. **A**, **B** 3-way ANOVA with mixed-effect model for repeated measurements, followed by original FDR method of Benjamini and Hochberg post-hoc multi-comparison test. **C**–**I** Unpaired 2-tailed Student’s tests with Welch’s correction. Data are presented as the median ± interquartile range. * indicates significant (*p* < 0.05) differences between genotypes (WT vs TG.^AC8^)

Significant changes in cytosolic LC3I and its lipidated product, LC3II (LC3-PE), recruited to the autophagosomal membrane during autophagy, were evident in TG^AC8^ vs WT. Specifically at 3–4 months, LC3AI was significantly upregulated in TG^AC8^ vs WT, whereas LC3AII was unchanged (Fig. 1A); in contrast, LC3BI did not change in TG^AC8^, whereas LC3BII was significantly downregulated (Fig. 1B), vs WT. Further, phosphorylation of LC3 at S12, which inhibits LC3 activity [24], was reduced by 50% (Fig. 1C), whereas phosphorylation of LC3 at T50, which enhances LC3 activity [25], was increased by 30% (Fig. 1D) in TG^AC8^ vs WT.

The expression of the essential autophagy protein adaptor [26] p62 was significantly upregulated in TG^AC8^ vs WT (Fig. 1E). In addition, protein levels of p62^S405^ (S403 human site) (Fig. 1F), which has increased affinity to ubiquitinated cargo [27], including depolarized mitochondria [28], and of p62^S349^ (Fig. 1G), which activates NRF2 pathway [29], were also significantly elevated in TG^AC8^ vs WT, as well as the ratios of phosphoproteins at both sites to total p62 (Fig. 1H, I).

Our previous work had shown that protein levels of lysosomal cathepsins are upregulated in the TG^AC8^ heart at 3–4 months of age [13]. Here, we quantified relative changes in different cathepsins in TG^AC8^ vs WT. Cathepsins Z and S were *more enriched* in TG^AC8^, whereas cathepsin L1 showed the least significant change (Supplemental Fig.  1A), vs WT. Because cathepsin L1 degrades not only lysosomal content but also autophagosome membrane components (included LC3II [30]) during the autophagic proteolysis, we assessed its activity as a measure of lysosome function and disposal of autophagic flux. Cathepsin L1 activity was significantly higher in TG^AC8^ vs WT (Supplemental Fig. 1B) in the context of an increase in the lysosomal acid phosphatase (ACP2) protein (Supplemental Fig.  1C).

**Fig. 2:**
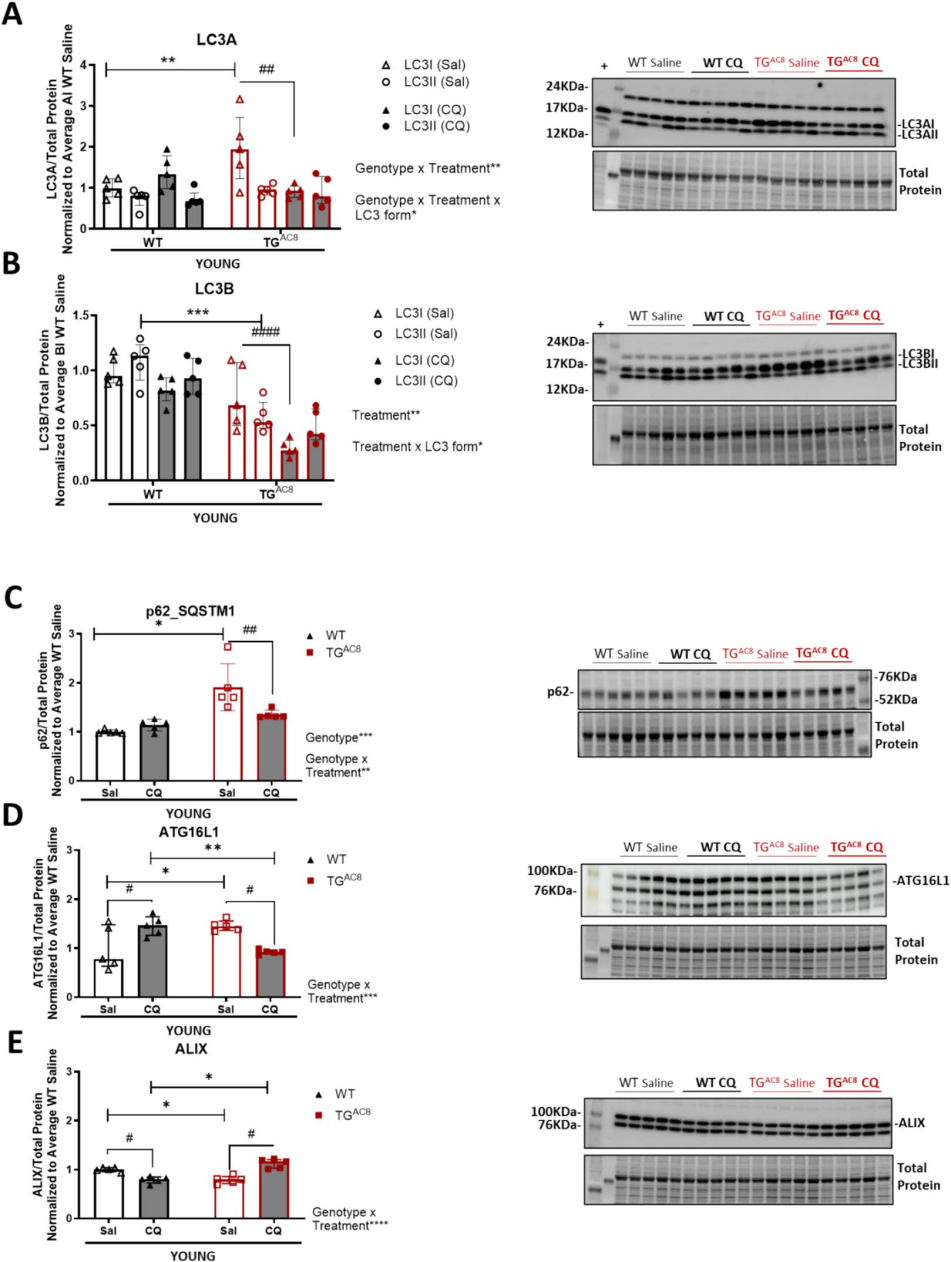

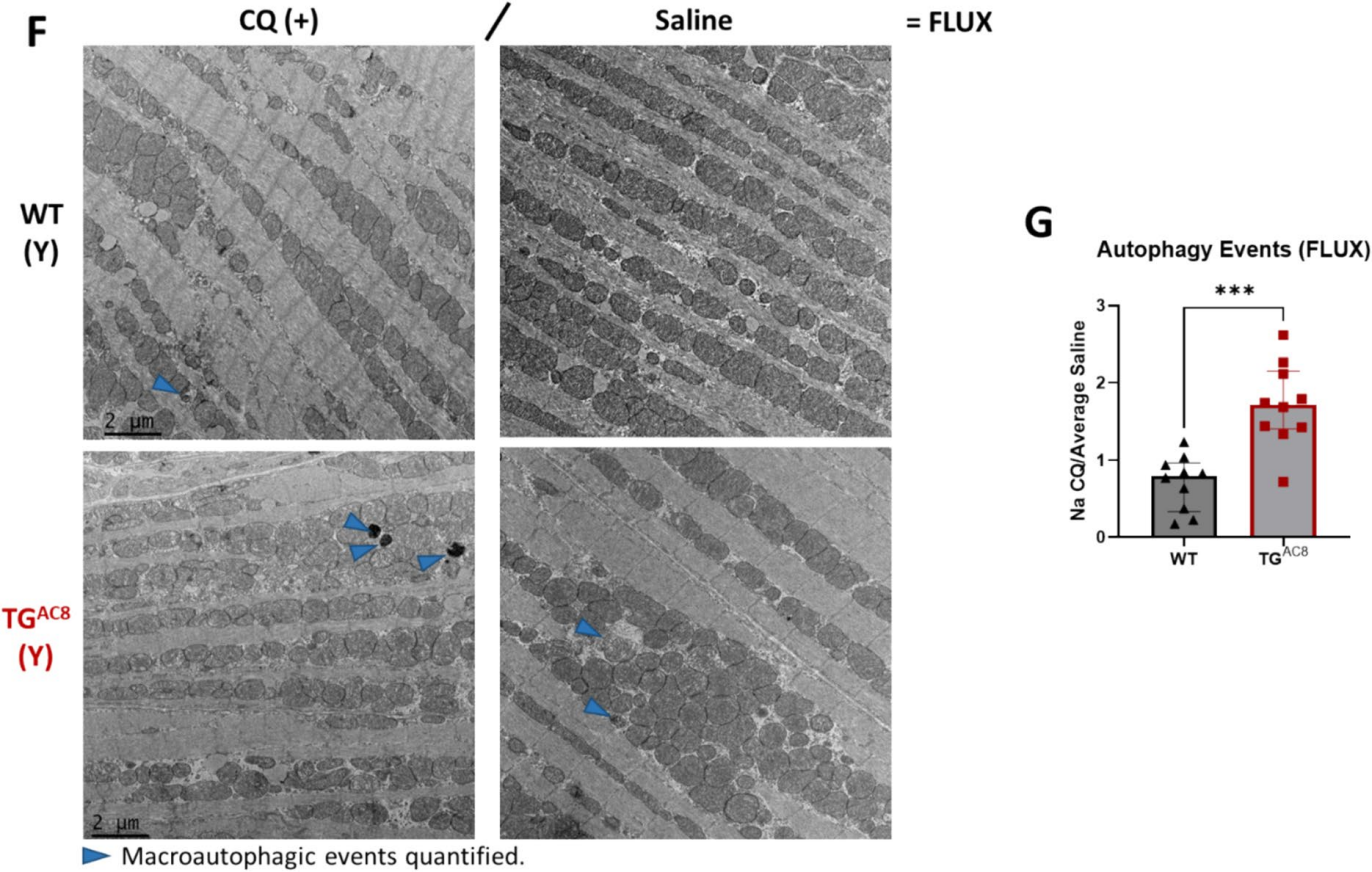
Autophagic flux is enhanced in TG^AC8^ at 3–4 months of age. Young TG^AC8^ and WT were treated by intraperitoneal administration (IP) with CQ (50 mg/kg) or saline, and LVs collected 3 h after and snap-frozen for analysis of autophagy markers (*n* = 5 mice/group). Bar graphs and WB of **A** LC3A, **B** LC3B, **C** p62, **D** ATG16L1, **E** ALIX, and **F** TEM representative micrographs and **G** paired quantification of macroautophagic figures, normalized to average WT-saline. **A**, **B** 3-way ANOVA with repeated measurements. **C**–**E** 2-way ANOVA. Original FDR method of Benjamini and Hochberg post-hoc multi-comparison test was used following both ANOVAs. **G** Unpaired 2-tailed Student *t* test with Welch’s correction. Data are presented as the median ± interquartile range. * and ^#^ indicate significant (*p* < 0.05) differences between genotypes* (WT vs TG^AC8^) and treatments.^#^ (CQ vs saline)

Because autophagy is a dynamic, multi-step process, we next evaluated the autophagic flux by inhibiting lysosomal function with the lysosomotropic drug chloroquine (CQ). Basal autophagy clearly differed in young TG^AC8^ vs young WT (Fig. 2A, B; Supplemental Fig. 2): autophagic “carrier-flux” (flux of the “carrier”-protein not the actual cargo/substrate-flux) of both LC3AI and LC3BI (Fig. 2A, B) became significantly reduced following CQ treatment, compared to saline, whereas for LC3AII and LC3BII, it remained unchanged; the carrier-flux of p62 (Fig. 2C) was also downregulated (by 29%) in TG^AC8^ vs WT, whereas the pattern of the carrier-flux of other important players involved in autophagy, following CQ, was more variable. Specifically, the carrier-flux of the *autophagy related 16 like 1* protein (ATG16L1), which participates in LC3-lipidation [31] and cell homeostasis by regulating membrane cellular trafficking [32], was reduced (by 37%) (Fig. 2D). In contrast, the carrier-flux of the *autophagy related 4B cysteine peptidase* (ATG4B) (Supplemental Figs. 3 lanes 1–12, 4A), which is involved in LC3-lifecycle [33] by processing pro-LC3 into LC3I [33], and of the *programmed cell death 6-interacting protein* (PDCD6IP/ALIX) (Fig. 2E), an ancillary protein contributing to the ESCRT signaling [34], were both increased (by 42% and 28%, respectively). Finally, the carrier-flux of the *peptidyl-prolyl cis–trans isomerase* (FKBP8) (Supplemental Fig. 4B), non-canonical mitophagy receptor [35] recruited to the mitochondria from LC3A [36]), did not change between genotype.

Using transmission electron microscopy (TEM), we next assessed events-flux by directly visualizing autophagic figures in saline- vs CQ-treated TG^AC8^ and WT. A significant increase in the numbers of autophagic events was detected in TG^AC8^ vs WT (Fig. 2F, G), confirming the increase in autophagy/autophagic flux in TG^AC8^ at this age.

Overall, chronic cardiac-specific over-expression of AC8 at 3–4 months of age [13] (1) modulates protein levels of the autophagic machinery by fine-tuning key markers of selective cargo recognition and of selective autophagy; (2) activates protection mechanisms against oxidative stress; and (3) enhances lysosomal degradation, ensuring a more efficient and effective clearance of misfolded proteins without accumulation of insoluble protein aggregates.

### Increased efficiency of autophagy/autophagic flux of the youthful TG^AC8 ^heart is lost in advanced age

**Fig. 3:**
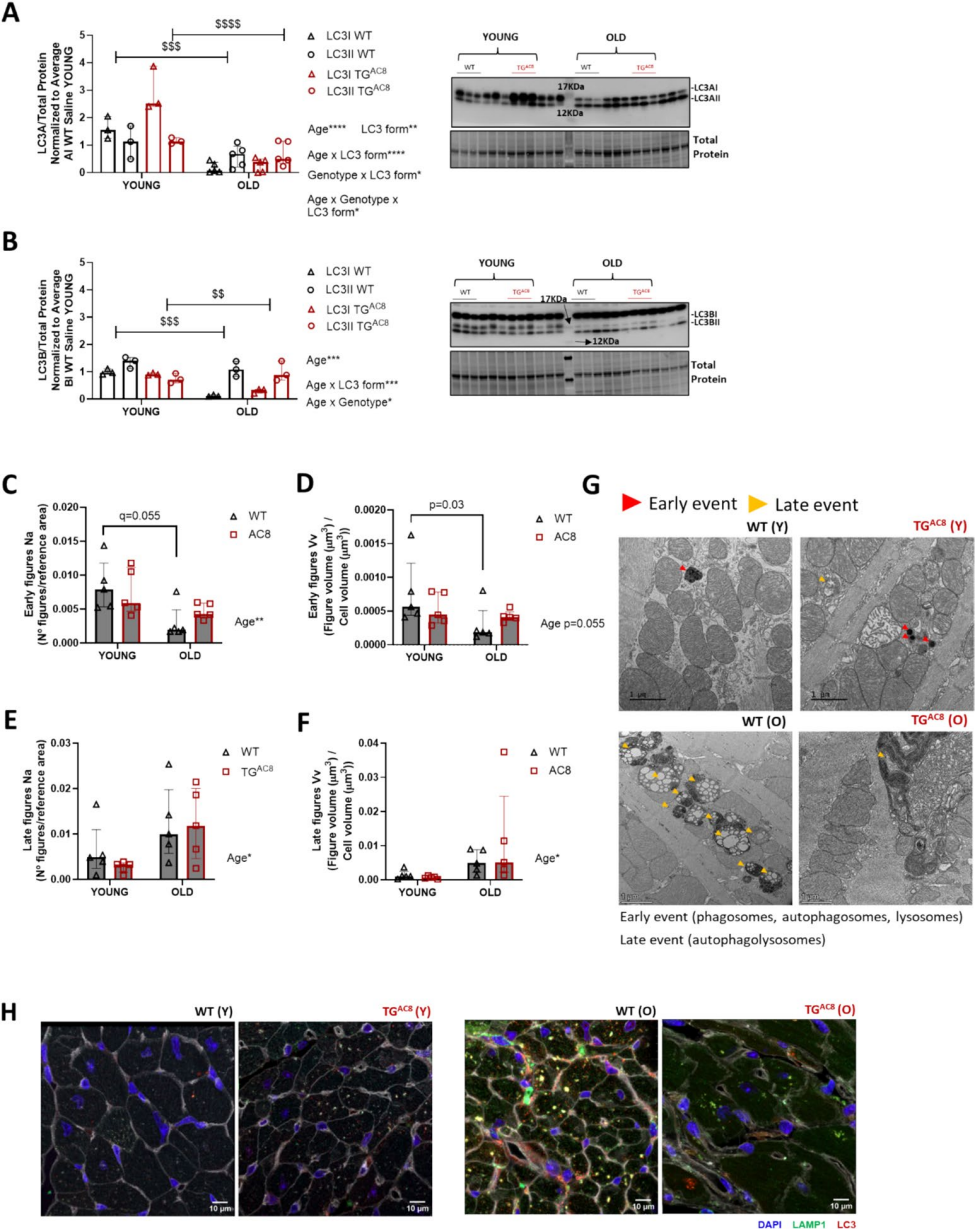

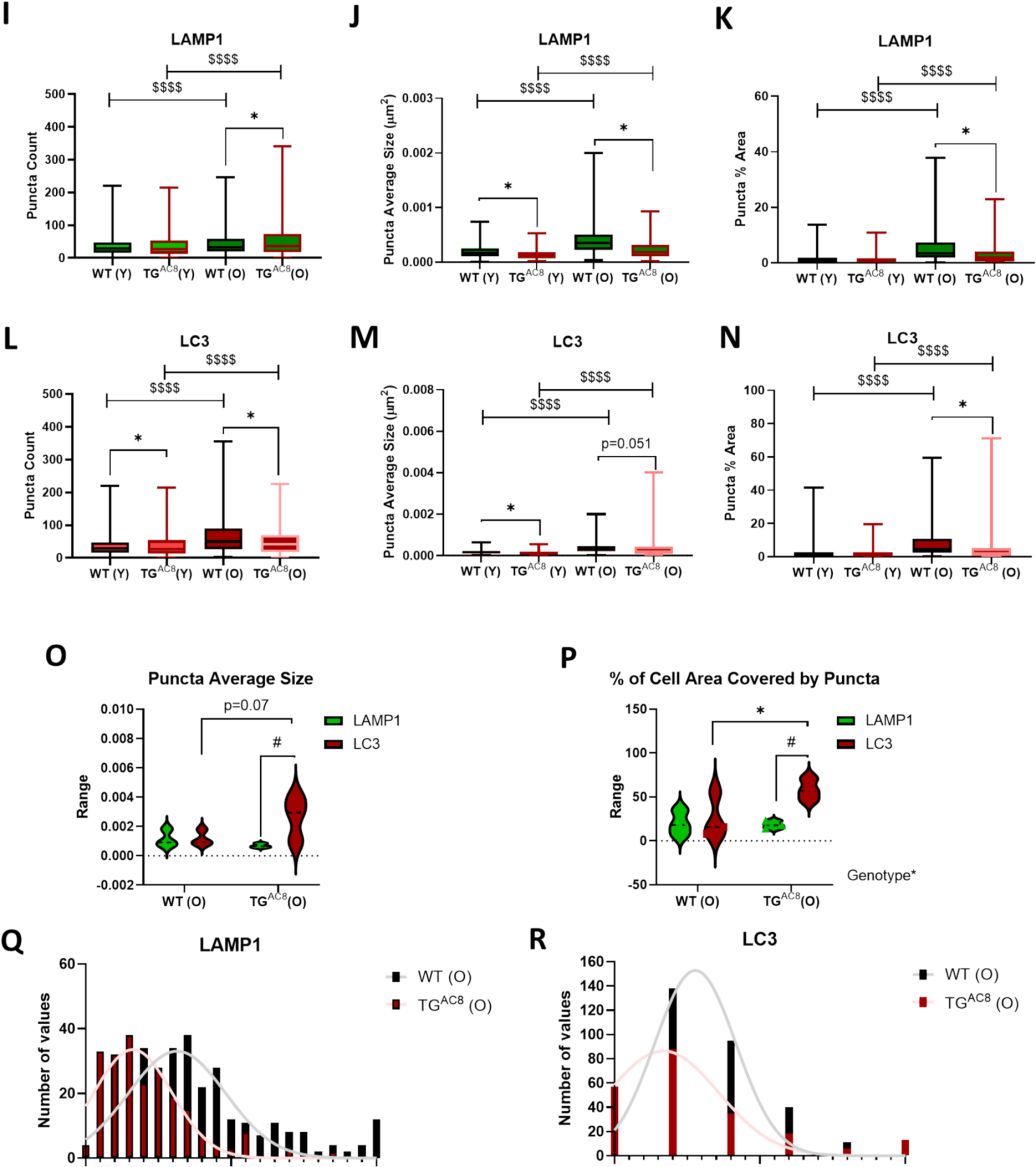
Autophagy failure in aged TG^AC8^ increases inclusions’ aggregation and size heterogeneity. **A**, **B** Bar graphs and WB of LC3A and LC3B and **C**–**F** bar graphs of macroautophagic figures (TEM) and **I**–**N** box and whiskers of endogenous LC3^+^-puncta (IHC, red) and LAMP1^+^-puncta (IHC, green) in young and old (*n* = 3–5 mice/group) TG^AC8^ and WT. **A** LC3A; **B** LC3B; **C** early-figures number/reference area; **D** early-figures volume/cell volume; **E** late-events number/reference area; **F** late-events volumes/cell volume; **G** TEM representative images; **H** IHC representative images; **I** LAMP1^+^-puncta count; **J** LAMP1^+^-puncta average size; **K** % of cell area covered by LAMP1^+^-puncta; **L** LC3^+^-puncta count; **M** LC3^+^-puncta average size; **N** % of cell area covered by LC3^+^-puncta; violin plots describing the **O** range of LAMP1^+^- and LC3^+^-puncta average size and of the **P** % of cell area covered by LAMP1^+^- and LC3^+^-puncta; **Q**, **R** LAMP1^+^-/LC3^+^-puncta size distribution in old TG^AC8^ and WT. **A**, **B** 3-way ANOVA with repeated measurements. **C**–**F**, **I**–**P** 2-way ANOVA. Original FDR method of Benjamini and Hochberg post-hoc multi-comparison test was used following both ANOVAs. Data are presented as the median ± range in **A**, and as median ± interquartile range in **B**–**E**. * and ^$^ indicate significant (*p* < 0.05) differences between genotypes* (WT vs TG^AC8^) and ages.^$^ (young vs old)

Because autophagy induction and clearance are known to decrease with aging [9], we anticipated that expression of key autophagic proteins and dissipated autophagic flux became reduced in aged TG^AC8^ and age-matched WT, compared to young animals. Indeed, LC3 (Fig. 3A, B) and ATG4B (Supplemental Figs. 3, 4A) were significantly downregulated in both genotypes at 17–21 vs 3–4 months of age, with specific accumulation of LC3II; in addition, the number/volumetric density of early autophagic figures (Fig. 3C, D, and G) were reduced, together with an increase in number/volumetric density of late autophagic figures (Fig. 3E–G), measured by paired TEM micrograph analysis (planimetry and stereology). Moreover, the number/size of endogenous LC3^+^-puncta (autophagosomes) and LAMP1^+^-puncta (lysosomes/late endosomes) (by fluorescence microscopy IHC) (Fig. 3H) were also significantly increased, with aging, independently of genotype (Fig. 3I–N). These data confirm the downregulation of autophagy induction and clearance with aging, as previously shown.

However, and interestingly, the aging process of TG^AC8^ differed from that in aging WT, in several aspects: (1) both LAMP1^+^- and LC3^+^-puncta were *smaller* in TG^AC8^, vs age-matched WT, at *both* ages (Fig. 3J, M); (2) although in *young* TG^AC8^, LC3^+^-puncta were *more numerous* (Fig. 3L) and there was no difference in the % of cell area they covered, vs WT (Fig. 3M), LC3^+^-puncta became *less numerous* (Fig. 3L) as the TG^AC8^ *aged*, vs WT, whereas LAMP1^+^-puncta counts significantly increased (Fig. 3I); in addition, in aged TG^AC8^, both LAMP1^+^- and LC3^+^-puncta covered a smaller area of the cell, independent of count, vs WT (Fig. 3K, N), but the latter were much more heterogeneous in size (Fig. 3O, p), and had a different frequency distribution (Fig. 3Q, R).

#### Assessment of autophagic flux

**Fig. 4:**
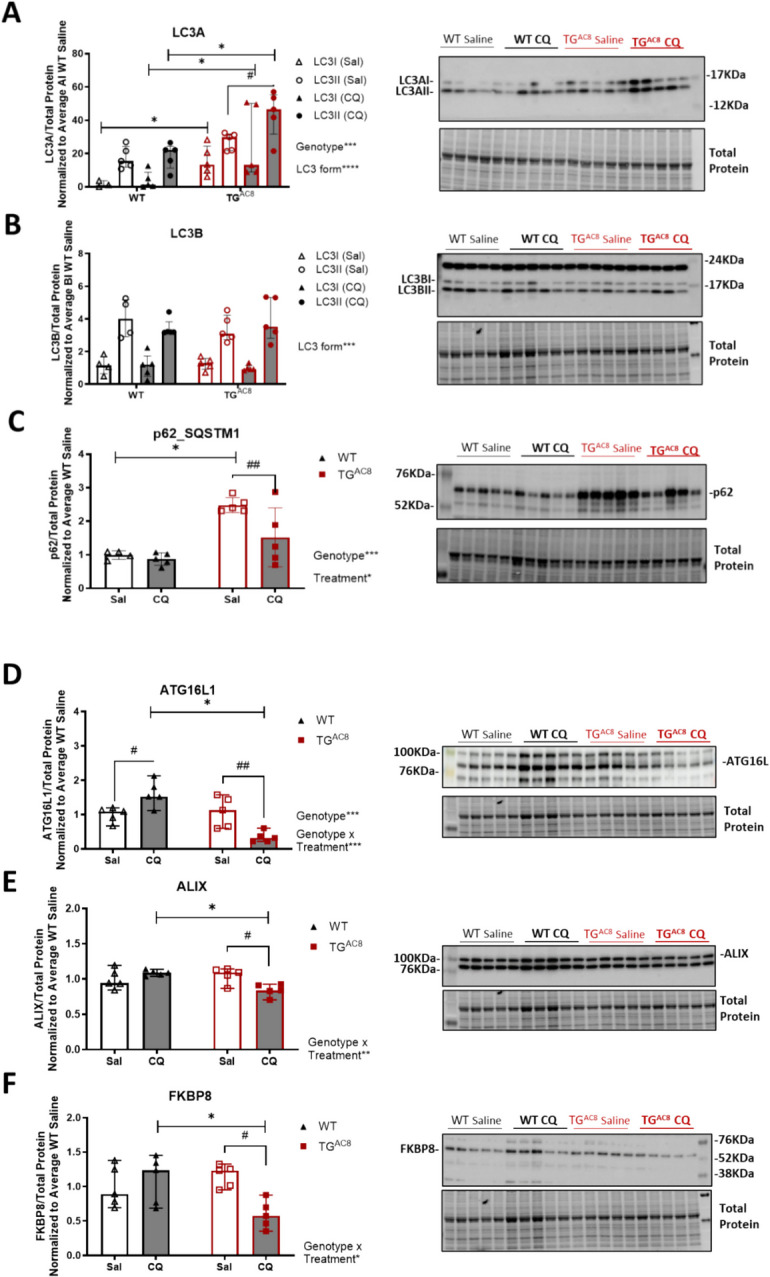

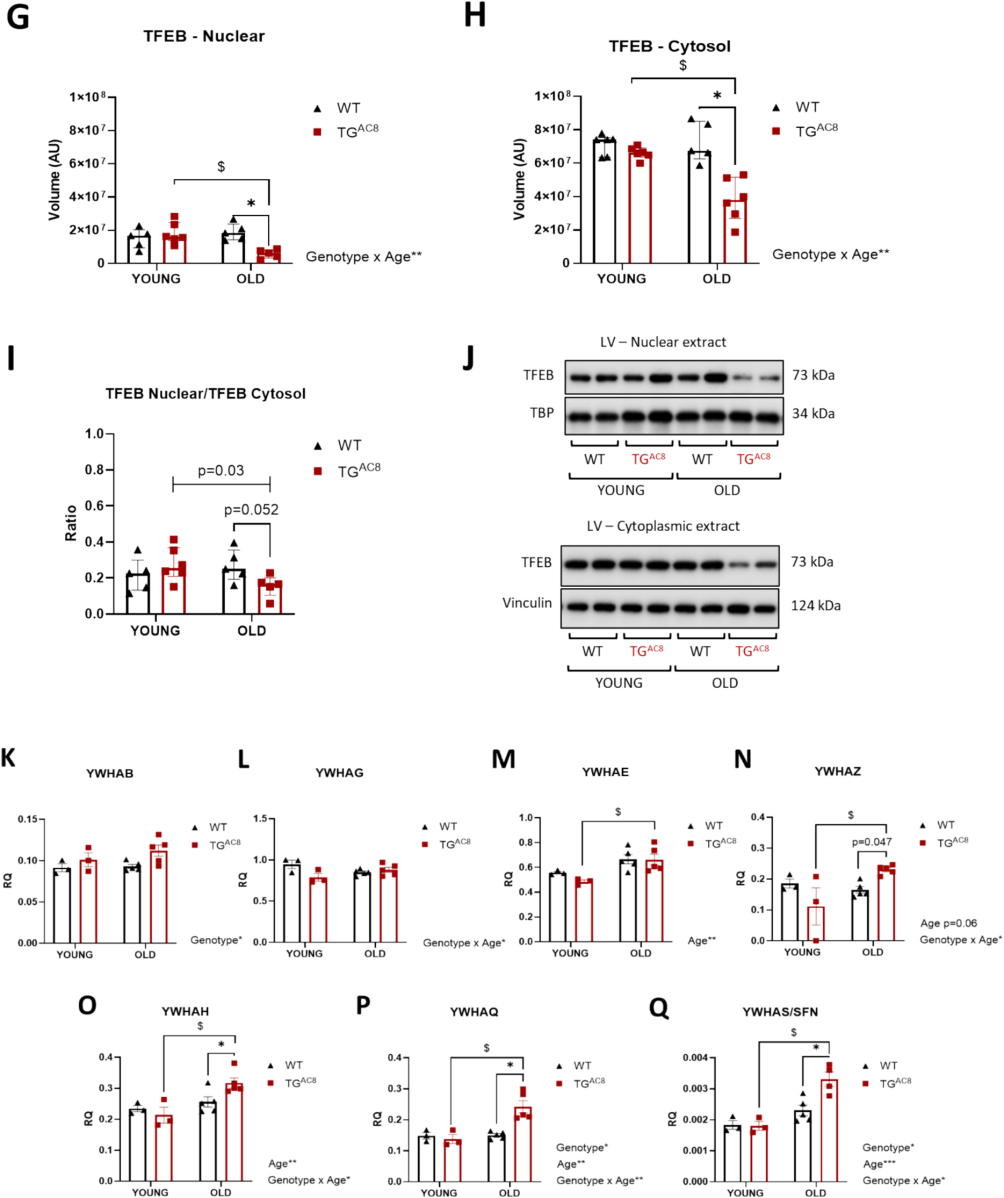
Accelerated autophagic flux in young TG^AC8^ heart becomes maladaptive and insufficient in old age, leading to more severe suppression of the autophagy program. **A**–**F** Old TG^AC8^ and WT were treated by intraperitoneal administration (IP) with CQ (50 mg/kg) or saline, and LVs (*n* = 5 mice/group) were collected 3 h after and snap-frozen for analysis of LC3, p62, ATG16L1, ALIX, FKBP8, and ATG4B. Bar graphs and WB analysis of **A** LC3A, **B** LC3B, **C** p62, **D** ATG16L1, **E** ALIX, and **F** FKBP8; **G**–**J** total TFEB protein levels in cytosolic and nuclear enriched fractions in young and old TG^AC8^ and WT (*n* = 5 mice/group). **G** Total TFEB in nuclear fraction; **H** total TFEB in cytosolic fraction; **I** TFEB nuclear/TFEB cytosol ratio; **J** representative WB images of nuclear and cytoplasmic extracts. **K**–**Q** Quantification of 14–3-3 s transcripts in young and old TG^AC8^ and WT (*n* = 3–5 mice/group). HPRT was used as housekeeping gene. Transcripts of **K** YWHAB, **L** YWHAG, **M** YWHAE, **N** YWHAZ, **O** YWHAH, **P** YWHAQ, and **Q** SFN. **A**, **B** 3-way ANOVA with repeated measurements. **C**–**I**, **K**–**Q** 2-way ANOVA, followed by original FDR method of Benjamini and Hochberg post-hoc multi-comparison test that was used following both ANOVAs. **A**–**I** Data are presented as the median ± interquartile range. **K**–**Q** Data are presented as the mean ± SEM. *, ^$^, and ^#^ indicate significant (< 0.05) differences between genotypes* (WT vs TG^AC8^), ages^$^ (young vs old), and treatments.^#^ (CQ vs saline)

Measurement of specific LC3 isoforms, p62, and assessment of autophagic flux (following CQ) (by WB) showed that LC3AI was significantly upregulated in old TG^AC8^, vs age-matched WT (Fig. 4A), similar to the genotypic difference at 3–4 months (Fig. 1A), whereas LC3AII protein levels did not significantly differ between genotypes at 17–21 months, *both* in the soluble- and insoluble-fraction/pellet (Fig. 4A, Supplemental Fig.  6A, C, respectively). In contrast, LC3B forms did not differ in aged TG^AC8^ vs WT (Fig. 4B), demonstrating that LC3 isoform specific modulation persists in old age (Fig. 1A, B). Similar to the younger age (Fig. 2E), also p62 protein levels in the soluble fraction were significantly upregulated in old TG^AC8^ vs WT (Fig. 4C); however, also *LC3AII-bound* p62 in the insoluble fraction (Supplemental Fig. 6B-C) showed greater accumulation in aged TG^AC8^ vs WT, an indication of increased presence of unprocessed LC3^+^-/p62^+^-aggregates/inclusions [37] in the old TG^AC8^ heart.

Following CQ treatment, both LC3AI and LC3AII significantly increased in aged TG^AC8^ vs age-matched WT-saline and WT-CQ (Fig. 4A); LC3AII also increased vs TG^AC8^-saline, an indication of an accelerated carrier-flux for LC3A in old TG^AC8^, vs WT. LC3B forms were not affected by CQ (Fig. 4B), whereas CQ remarkably reduced p62 protein levels in old TG^AC8^ in the soluble fraction (Fig. 4C), similar to younger age (Fig. 3C). The carrier-flux of ATG16L1 (Fig. 4D), ALIX (Fig. 4E), and of FKPB8 (Fig. 4F) were also *all significantly reduced* in old TG^AC8^ (by 68%, 20%, and 49%, respectively), an indication that *overall* autophagic flux in aged TG^AC8^ was significantly reduced, compared to both younger TG^AC8^ and to aging WT. In contrast, CQ treatment did not affect ATG4B carrier-flux in aged TG^AC8^ vs WT (Supplemental Fig. 7), and neither LC3 phosphorylation (S12 and T50) nor ACP2 protein levels (Supplemental Fig. 8A-C) differed by genotype in untreated aged animals.

#### TFEB phosphorylation

Because the *Coordinated Lysosomal Expression and Regulation* (CLEAR) network [38] orchestrates upstream signaling pathways of autophagy and lysosome biogenesis/function [39], we assessed protein levels and activation (by phosphorylation) of its master regulator, the transcription factor TFEB, in young and old TG^AC8^ and age-matched WT (Supplemental Figs. 9A-D). We observed a significant increase in the phosphorylation at S211 (TFEB^S211^), together with an increase in the ratio of TFEB^S211^ to total TFEB, with aging, in both genotypes (Supplemental Figs. 9A-D), an indication of the age-related decrease in autophagy induction, independent of genotype. However, and remarkably, cytoplasmic and nuclear enrichment of LV tissue showed that the nuclear to cytoplasmic ratio of total TFEB was significantly reduced in aged TG^AC8^ vs old WT (Fig. 4G–J). TFEB phosphorylation at S211 (by mTORC1 [40]) increases its interaction with 14–3-3 proteins in the cytoplasm, which prevents its translocation to the nucleus [40], consequently turning off the activation of TFEB downstream pathways. Because the 14–3-3, a family of ubiquitously expressed adaptor proteins, typically binds to “client proteins” at phosphorylated serine/threonine motifs (TFEB included), regulating their stability, activity, and/or localization [41], we then assessed 14–3-3 s’ transcriptional expression (by qPCR) in both young and old TG^AC8^ and age-matched WT. Five (YWHAE, YWHAZ, YWHAH, YWHAQ, and Stratifin) of the 7 14–3-3 s’ isoforms were significantly increased in aged vs young TG^AC8^, whereas 14–3-3’s transcripts did not change in WT with age (Fig. 4K–Q). Additionally, mRNAs of YWHAZ, YWHAH, YWHAQ, and Stratifin were also significantly increased in aged TG^AC8^ vs age-matched WT (Fig. 4N–Q). Transcriptional upregulation of 14–3-3 s together with reduced levels of *nuclear* TFEB explain the greater reduction in the activation of the CLEAR network in old TG^AC8^ vs WT hearts.

### Proteasome insufficiency and mitochondrial dysfunction lead to an exaggerated age-associated increase in aggregates’ accumulation in aged TG^AC8^

We next assessed PQC mechanisms (UPS activity, autophagy/mitophagy) and evaluated protein translation rates and aggregates accumulation in aged TG^AC8^ and age-matched WT. Our previous work in young mice (3–4 months of age) demonstrated that (1) UPS activity, autophagy, and mitophagy were all upregulated in TG^AC8^ vs WT, indicating cooperation among PQC mechanisms [13]; (2) although the protein synthesis rate was 40% higher in TG^AC8^ vs WT, UPS clearance of aggregates was also upregulated, and insoluble aggregates did not accumulate within cells [13]; (3) the numbers of healthy mitochondria and the % of cell volume these occupied did not differ between TGAC8 and WT [13]; and (4) although the canonical cargo receptor PARKIN was significantly upregulated, mitochondrial fitness was comparable between genotypes [13].

#### Proteasome activity and protein aggregates

**Fig. 5:**
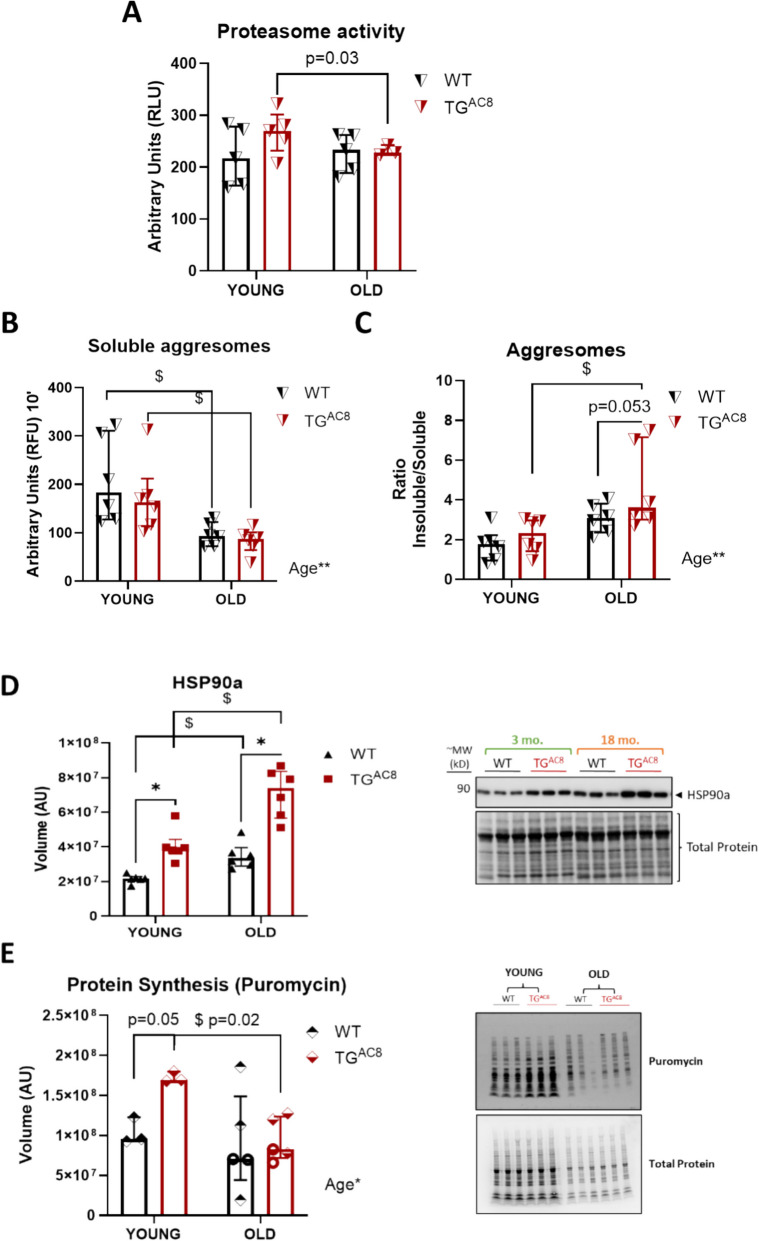
Proteostasis dysregulation is more severe in the old TG^AC8^ heart. Bar graphs of **A** proteasome activity assessment and **B**, **C** aggregates quantification in soluble fractions and the ratio of insoluble vs soluble protein aggregates in young and old TG^AC8^ and WT (*n* = 3–5/group). **D** Bar graphs and WB of HSP90α. **E** Old TG^AC8^ and WT (*n* = 5 mice/group) were treated by intraperitoneal administration (IP) with puromycin (0.022 g/g mouse) according to the SUnSET method, and LVs were collected 30 min after and snap-frozen for puromycin analysis. **A**–**E** 2-way ANOVA, followed by original FDR method of Benjamini and Hochberg post-hoc multi-comparison test. Data are presented as the median ± interquartile range. * and ^$^ indicate significant (< 0.05) differences between genotypes* (WT vs TG^AC8^) and ages.^$^ (young vs old)

Proteasome activity was not increased in old TG^AC8^, vs WT, but progressively declined in TG^AC8^ with aging, from 3 to 18 months, whereas it remained the same in WT (Fig. 5A). The density of *soluble* misfolded proteins also decreased with aging, but this reduction was independent of genotype (Fig. 5B). The ratio of insoluble to soluble aggregates, however, significantly increased in TG^AC8^ between 3 and 18 months, with no change in aging WT (Fig. 5C), indicating the greater accumulation of *insoluble* aggregates in the TG^AC8^ heart, during the aging process, compared to “normal” aging WT.

Because accumulation of *insoluble* aggregates leads to proteotoxic stress [42], we assessed protein levels (by WB) of *heat shock protein 90* (HSP90), a chaperon/stress-sensor that activates protective mechanisms [43] to reduce stress and maintain cellular homeostasis and intracellular transport [44]. The stress-inducible form HSP90α, significantly increased between 3 and 18 months in both genotypes (Fig. 5D), an indication that age, per se, increases cellular stress, as well as the need for increased protein folding/refolding/maintenance/degradation. Additionally, HSP90α was significantly upregulated in TG^AC8^ vs WT at both ages (Fig. 5D) but was increased to a further extent in old TG^AC8^, in parallel with the highest level of proteotoxic stress (Fig. 5C), and the persistent higher cAMP-derived chronic stress of the TG^AC8^ heart, compared to “normal’ aging WT. Although protein translation rates did not differ by genotype in aged mice (Fig. 5E), they significantly decreased in TG^AC8^ from 3 to 18 months, a hallmark of aging in health [45].

#### Mitochondrial dynamics

**Fig. 6:**
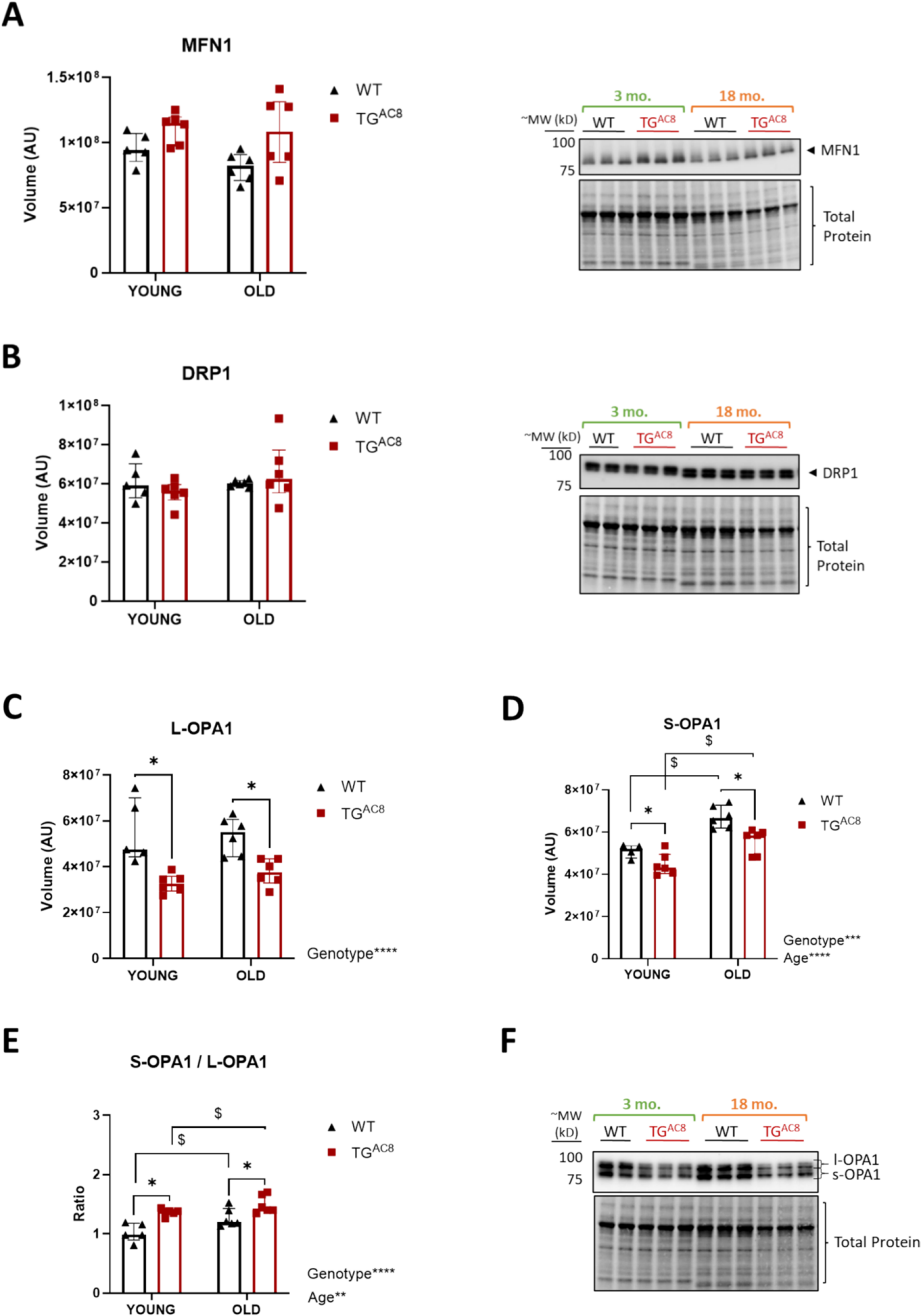

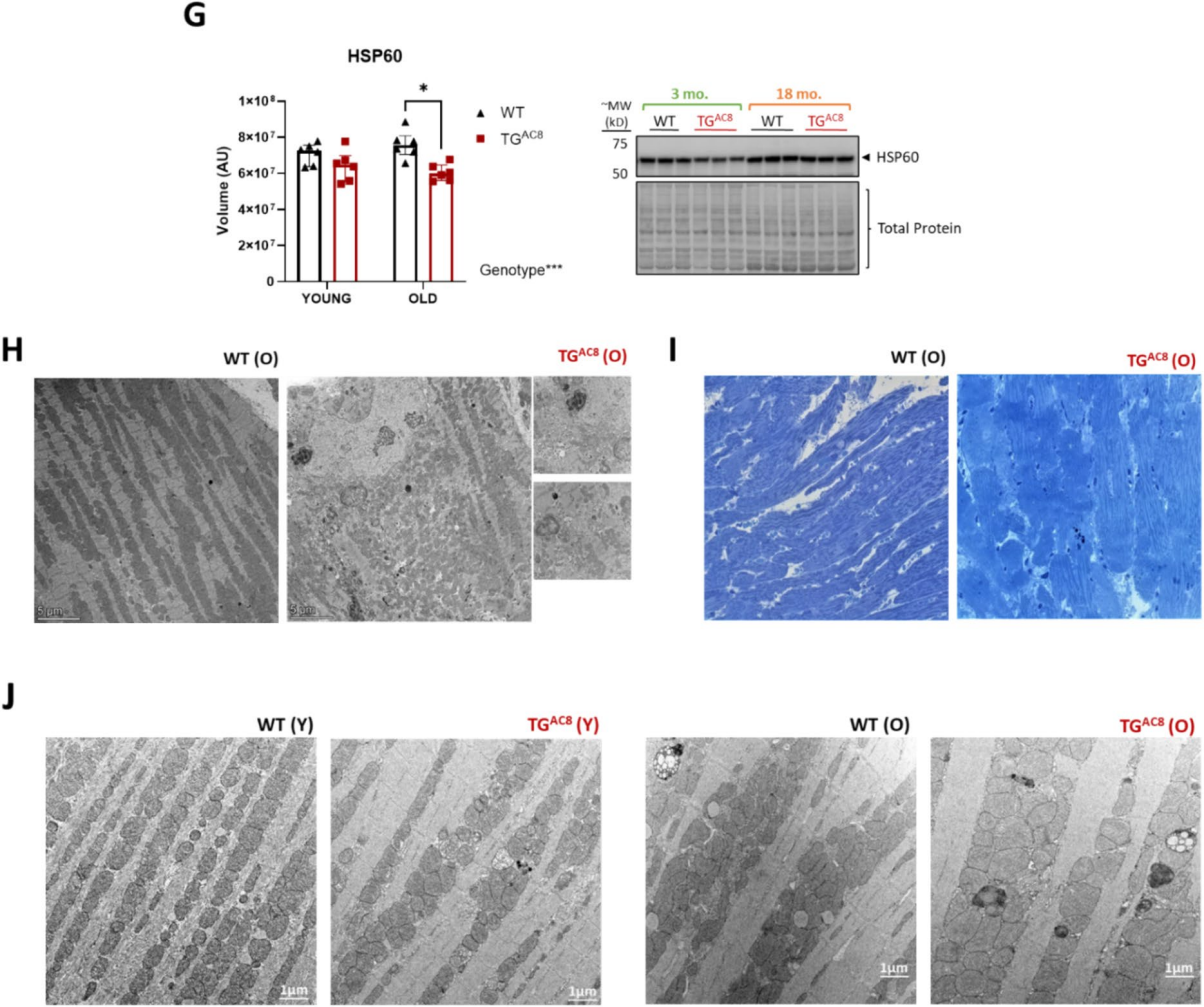

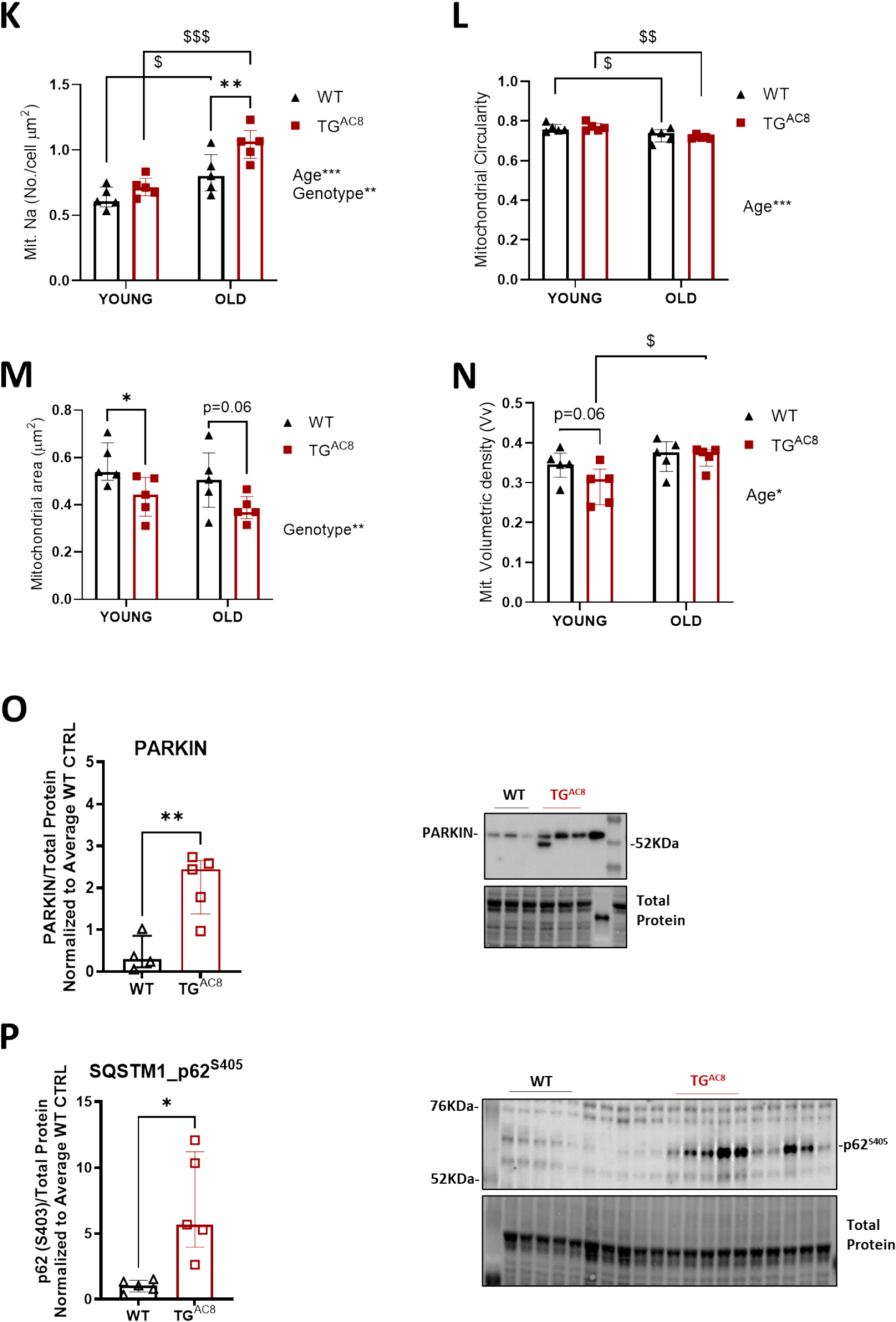

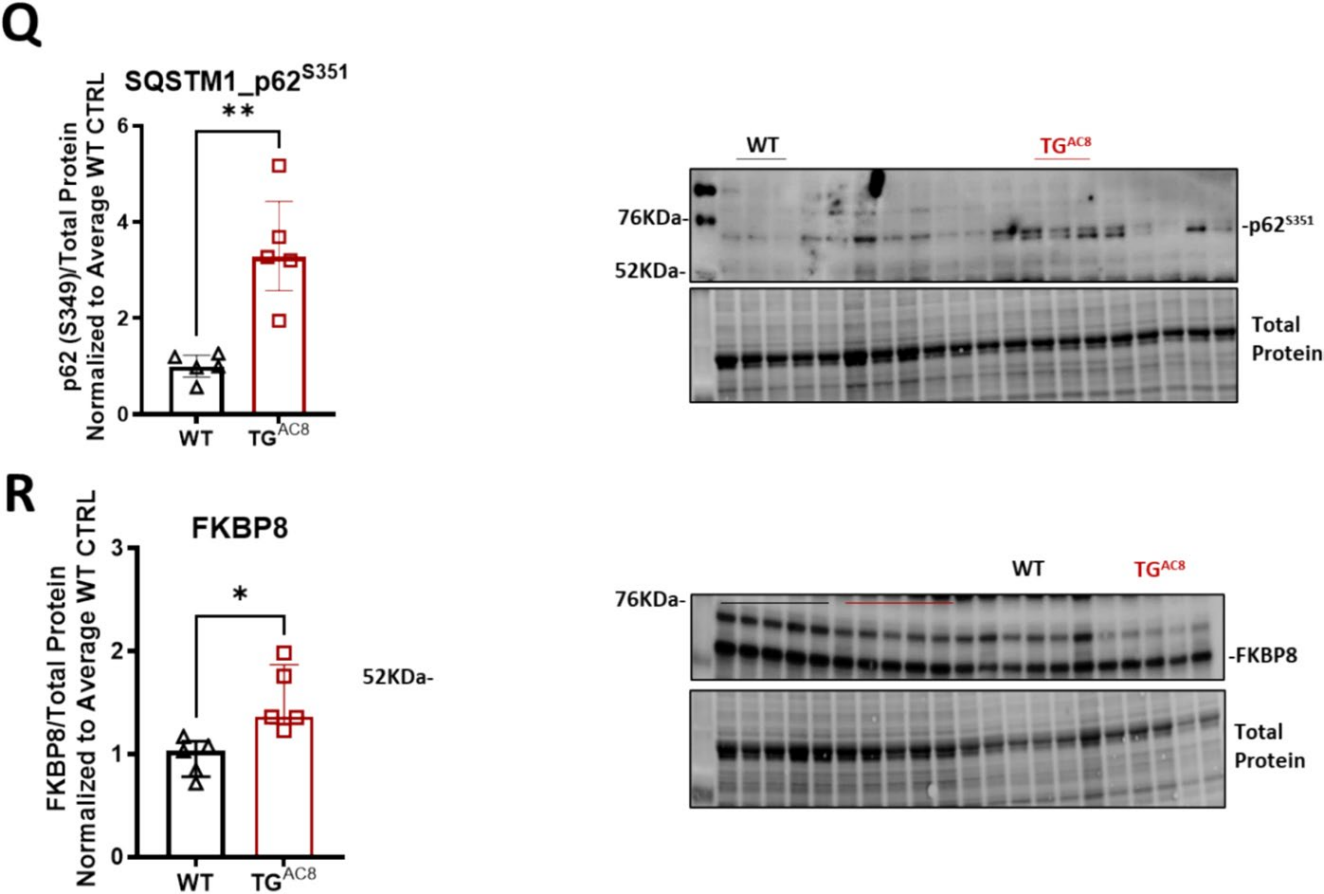
PQC insufficiency leads to mitochondria dysfunction and disruption of the mitochondrial network in old TG^AC8^ heart. **A**–**G**, **Q**–**T** Bar graphs and WB of mitochondrial markers; **H**, **I** representative TEM and light microscopic images in young and old TG^AC8^ and WT (*n* = 5 mice/group). **L**–**P** Quantification of mitochondrial parameters the same TEM images (15/animal, 300 in total), using a custom algorithm. **A** MFN1; **B** DRP1; **C** L-OPA1; **D** S-OPA1; **E** S-OPA/L-OPA1 ratio; **F** representative WB; **G** HSP60; **H** representative TEM and **I** light microscopy images of semi-thin sections showing disrupted mitochondrial network; **J** representative TEM images in young and old TG^AC8^ and WT; **K** mitochondrial number/area; **L** circularity; **M** mitochondrial area; **N** mitochondrial Vv; **O** PARKIN; **P** p62^S405^; **Q** p62^S349^; **R** FKBP8. **A**–**E**, **G**, **K**–**N** 2-way ANOVA, followed by **A**–**E**, **G** original FDR method of Benjamini and Hochberg and **K**–**N** Fisher’s LSD post-hoc multi-comparison test. **O**–**R** Unpaired 2-tailed Student *t* test with Welch’s correction. Data are presented as the median ± interquartile range for **A**–**E** and **G** and as the mean ± SD for **K**–**N**. * and ^$^ indicate significant (< 0.05) differences between genotypes* (WT vs TG^AC8^) and ages.^$^ (young vs old)

Mitochondrial failure, another hallmark of aging, occurs in diverse pathologic disease conditions, including HF [46], in response to a wide variety of stressors. We evaluated protein levels (by WB) of key players of mitochondrial dynamics (fusion/fission [47], function [48], integrity [49], and clearance [50]) in young and aged TG^AC8^ and age-matched WT. *Mitofusin-1* (MFN1), located on the outer mitochondrial membrane (OMM) and involved in mitochondrial fusion, was significantly increased in old TG^AC8^ (Fig. 6A), a scenario compatible with more favorable conditions for mitochondrial fusion and docking [51], vs old WT; however, levels of the mitochondrial GTPase *dynamin-related protein 1* (DRP1), a cytosolic protein that controls late stages of the mitofission process [52] and is recruited to the mitochondrial surface in response to various physiological cues [53], did not differ between genotypes (Fig. 6B). In contrast, levels of the protein *dynamin-related GTPase optic atrophy type 1* (OPA1), which is localized on the outer leaflet of the inner membrane (IMM) [54], differed in TG^AC8^ vs WT. The expression of both the *full-length* OPA1 (L-OPA1), involved in IMM fusion and cristae remodeling [55], and of its *cleaved-form* (S-OPA1), which is associated with mitochondrial fission [56] and results from the proteolytic processing of L-OPA1 (constitutively, by YME1L [57], and upon stress-activation, by OMA1 [58]), were significantly reduced in TG^AC8^ vs WT independent of age (Fig. 6C, D, and F). Remarkably, although TG^AC8^ expressed less S-OPA1 vs age-matched WT (Fig. 6D, F), the ratio of S-OPA1 to L-OPA1 was significantly elevated in TG^AC8^ vs WT at both ages (Fig. 6E) and was increased to a further extent in aged TG^AC8^ vs age-matched WT. Protein levels of the mitochondrial heat-shock protein 60 (mtHSP60/HSPD1), an indispensable chaperonin that regulates mitochondrial protein homeostasis/function by preventing the aggregation of misfolded proteins [59], were also reduced in aged TG^AC8^ vs WT (Fig. 6G).

#### Planimetric and stereological analysis of mitochondrial parameters

The structure of the mitochondrial network was more severely disrupted in old TG^AC8^ vs age-matched WT (Fig. 6H, I) (by TEM and light microscopic evaluation of semi-thin sections of LV-CMs). In line with these results, planimetric and stereological analysis of mitochondrial parameters, calculated by using an artificial intelligence (AI) segmentation-algorithm trained on the same TEM image set (Fig. 6J) in young and aged TG^AC8^ and age-matched WT, showed that the *number of individual mitochondrial cross-sections* per cardiomyocyte area (Fig. 6K) *was increased* and displayed *reduced circularity* (Fig. 6L), with aging, independently of genotype, an indication of the presence of a more convoluted shape of the mitochondrial network with aging, per se.

Of interest, although mitochondrial *cross-sectional areas* within the mitochondrial network were *smaller* (Fig. 6M) and *rounder* (Fig. 6N) in young TG^AC8^, and their volumetric density had a trend toward smaller values, compared to young WT, *cross-section circularity was further reduced* in old TG^AC8^ vs age-matched WT and was accompanied by a further increment of the individual cross-sections per area within an equivalent volumetric density, a scenario of increased mitochondrial fragmentation in aged TG^AC8^ vs old WT.

#### Expression of specific cargo receptors involved in canonical and non-canonical mitophagy

Because the mitophagy process ensures disposal of malfunctional and/or dysfunctional mitochondria, we next evaluated the expression of specific cargo receptors involved in canonical and non-canonical mitophagy [60] in aged TG^AC8^ and age-matched WT. Similar to young TG^AC8^, the canonical cargo receptors PARKIN and p62^S405^ (Fig. 6O, P), recruited to depolarized [61] and polyubiquitinated [28] mitochondria, respectively, and p62^S349^ (Fig. 6Q), upregulated in conditions of oxidative stress [29], were *all* significantly increased in aged TG^AC8^ vs WT. In contrast to young TG^AC8^ (Supplemental Fig. 4B), the non-canonical cargo receptor FKBP8 [62], which assists LC3A during mitophagy and in the clearance of misfolded proteins, was also significantly upregulated in aged TG^AC8^ vs old WT (Fig. 6R).

#### Lipofuscin

**Fig. 7:**
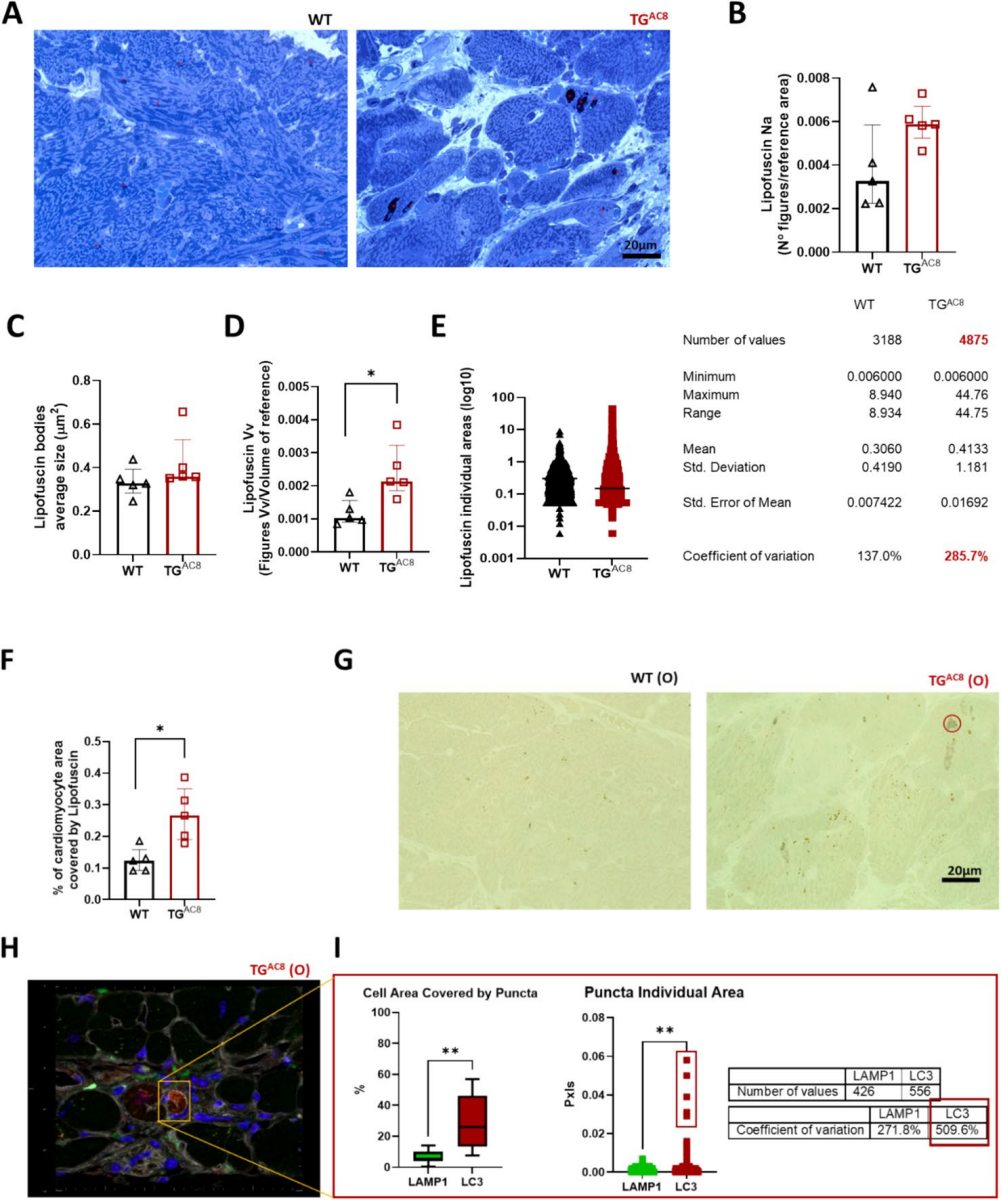

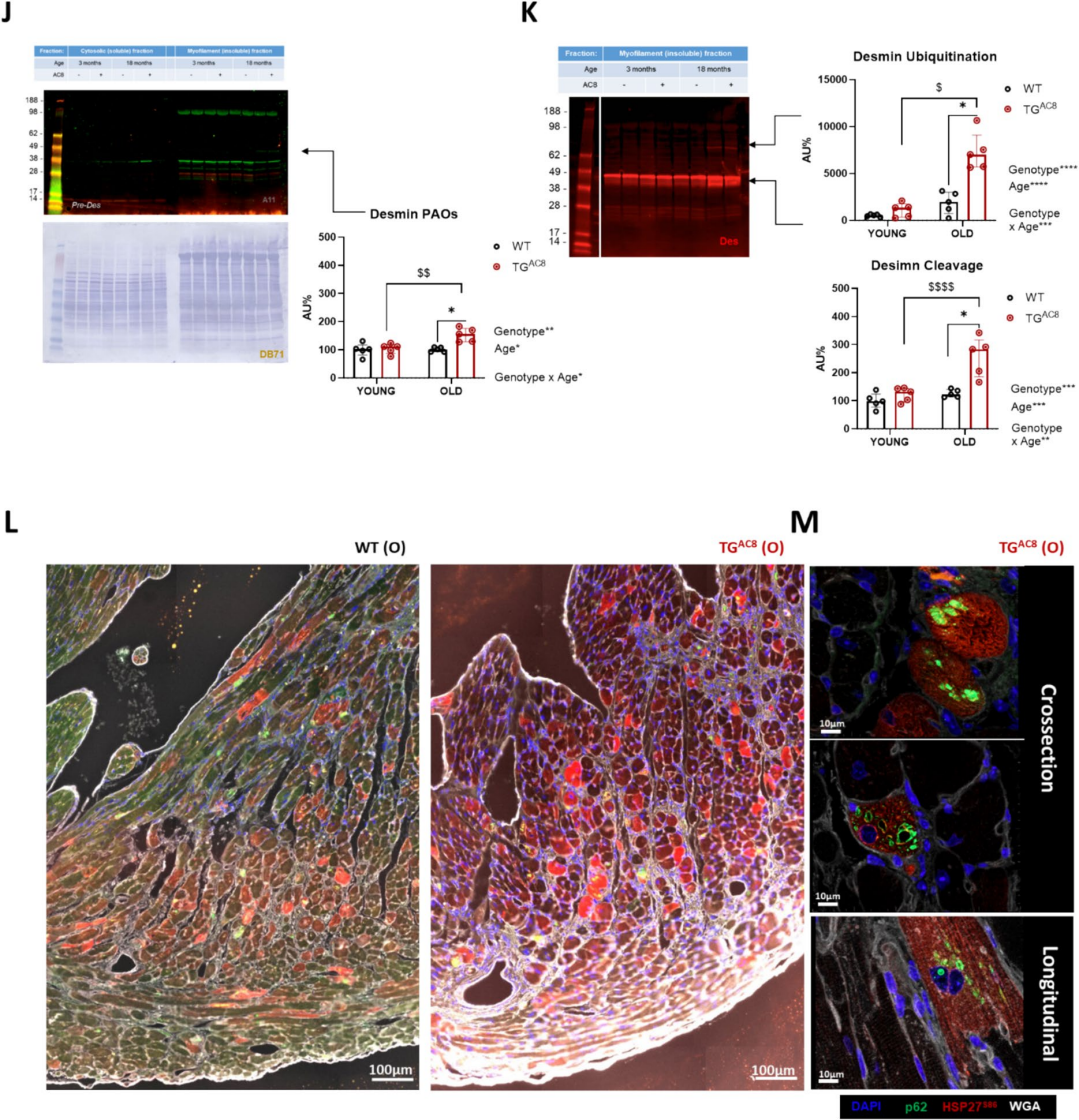
Proteostasis dysregulation from PQC insufficiency leads to increased lipofuscin accumulation and deposition of desmin-PAOs in aged TG^AC8^. Bar graphs representing the quantification of undigested material (LF, desmin and LC3^+^- and LAMP1^+^-inclusions) in old TG^AC8^ and WT (*n* = 5 mice/group); **A** representative semi-thin staining of LF bodies; **B** number of LF figures/reference area; **C** average size of LF bodies; **D** volume of LF figures/volume of reference area; **E** violin plot showing the more aberrant sizes of LF bodies; **F** percentage of cardiomyocyte area covered by LF; **G** representative image of Palade staining of TEM semi-thin sections showing the presence of brown-to-blueish/black pigments in old TG^AC8^; **H** representative image of IHC sections showing cardiomyocytes covered with LC3^+^-inclusions in old TG^AC8^; **I** quantification of cell area covered by LAMP1^+^- and LC3^+^-inclusions in old TG^AC8^ and box and whiskers describing the quantification of LAMP1^+^- and LC3^+^-puncta individual areas in old TG^AC8^; **F** soluble and insoluble (myofilament-enriched) fractions probed with the A11 antibody for PAOs (top), and total protein staining with Direct Blue71/DB71 (bottom), with relative densitometry analysis; **J** insoluble (myofilament-enriched) fractions probed with the desmin antibody and **K** relative densitometry for ubiquitinated and cleaved desmin proteoforms; **L**, **M** representative IHC staining of HSP27^S82^ (red), p62 (green), and wheat germ agglutinin WGA (white) in old TG^AC8^ and WT (nuclei are in blue (DAPI); **B**–**F**, **I** unpaired 2-tailed Student *t* test with Welch’s correction. **J**, **K** 2-way ANOVA, followed by original FDR method of Benjamini and Hochberg post-hoc multi-comparison test. Data are presented as the median ± interquartile range. * and ^$^ indicate significant differences (*p* < 0.05) between genotypes* (WT vs TG^AC8^) and ages.^$^ (young vs old)

LF body quantification (in Palade’s-stained semi-thin sections by light microscopy) (Fig. 7A) showed that there was no difference in LF number or average body size (Fig. 7B, C) in old TG^AC8^ vs age-matched WT. However, there was a *marked heterogeneity* within the LF population and *increased total volume of LF* (Fig. 7D, E), with LF covering a larger percentage of the cardiomyocyte area (Fig. 7F), in aged TG^AC8^ vs old WT. Interestingly, those vast insoluble aggregates (reminding a myelin-like figure with high electron-dense concentric lamellations) displayed different pigmentation (brownish to blueish-black) (Fig. 7G). Additionally, assessment of LC3^+^-autophagic-cargo (by IHC) demonstrated the presence in old TG^AC8^, but not in old WT, of LC3^+^-inclusions that were very heterogenous in size (Fig. 7H) and almost entirely covered the cardiac myocyte (CM) cell area (LC3^+^-CMs) (Fig. 7I).

#### Accumulation of PAOs and HSP27

Recent evidence has shown the involvement of desmin, whose filaments interlink the contractile myofibrillar apparatus to mitochondria, nuclei, and sarcolemma, to impaired mitochondrial fission and proteostasis [63]. Furthermore, desmin disorganization has been documented to be a component of the accumulation of PAO-aggregates in the heart [64]. Desmin-PAOs increased with age in TG^AC8^ but not in WT and were higher in old TG^AC8^, vs age-matched WT (Fig. 7J). Further, a higher percentage of desmin was tagged for ubiquitination and cleaved (Fig. 7K), an indication of severe alterations of the myofilament ultrastructure in the old TG^AC8^ heart vs WT.

HSP27^S82^, the stress-induced phospho-form (by MAPK [65]) of the small heat shock protein (sHSP) HSP27/HSPB1, a biomarker of cardiac damage [66] that is upregulated during stress conditions to maintain myocardial function [67], was significantly elevated in old TG^AC8^ vs age-matched WT (Fig. 7L) and specifically co-localized (via fluorescence microscopy IHC) with p62^+^-inclusions (Fig. 7M), an indication of higher stress and increased myofilament disruption, exactly at the site of electron-dense aggregate accumulation, in aged TG^AC8^- vs old WT-CMs.

### Changes in cardiac structure/function

#### Increased failure of PQC mechanisms exacerbates cardiac aging in TG^AC8^

**Fig. 8:**
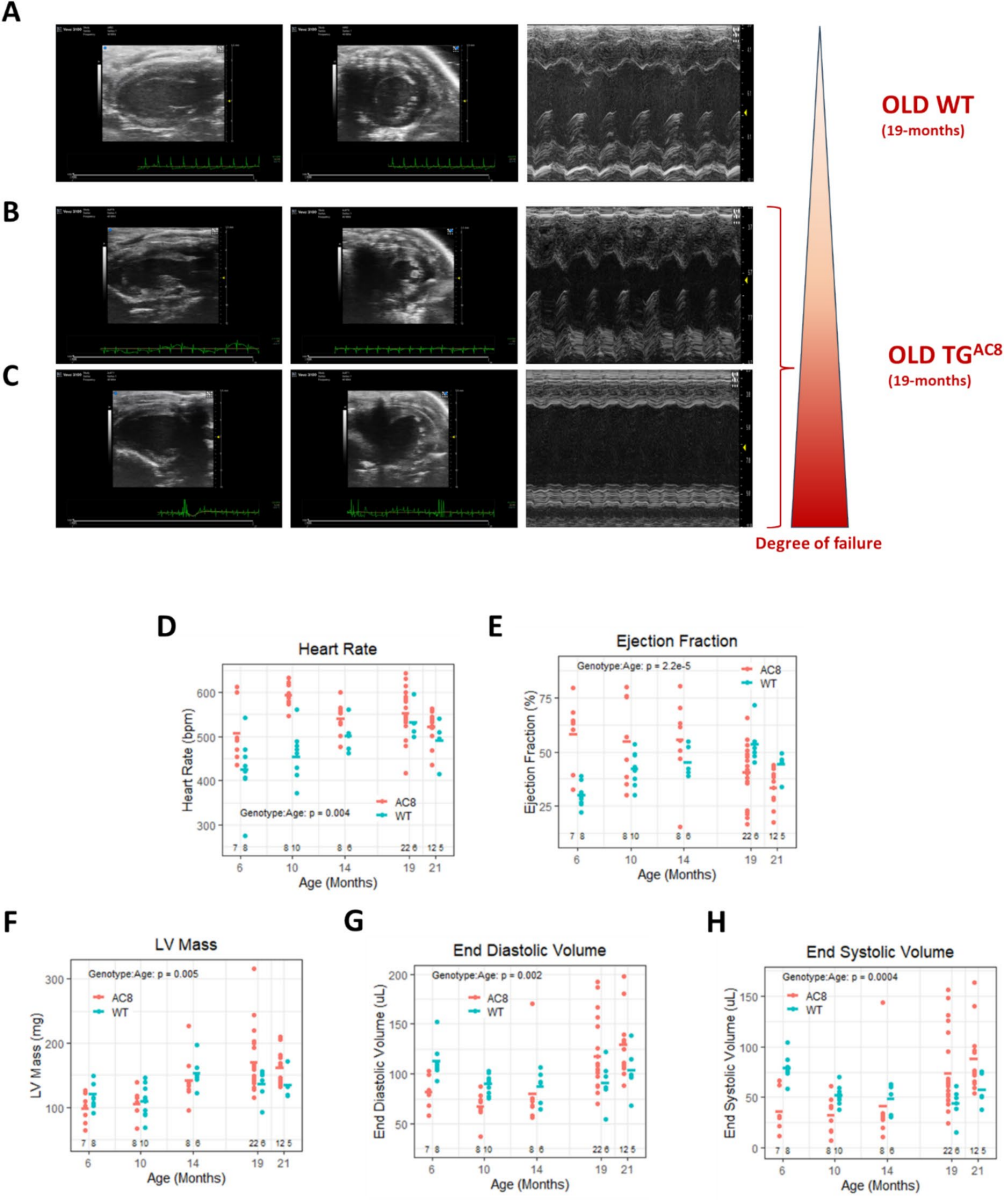
Heart damage by proteotoxic stress and mitochondrial dysfunction is associated with heart failure. **A**–**C** Representative images of echocardiograms in old TG^AC8^ and WT (*N* = 6–15 mice/group). Echocardiographic parameters **D** heart rate (HR), **E** ejection fraction (EF), **F** left ventricular mass (LVM), **G** end diastolic volume (EDV), and **H** end systolic volume (ESV). Data are presented as mean ± SEM. Differences among groups were assessed by ANOVA

We next addressed what changes occurred in cardiac structure/function in the context of dysregulated proteostasis. To this end, assessment of cardiac function and structure by echocardiogram revealed that the small hyperdynamic heart of TG^AC8^ at 3–4 months of age (significantly smaller LV chamber size with higher ejection fraction (EF) and heart rate (HR), compared to young WT) [13] became a hypertrophic and dilated heart at 19 months (Fig. 8A–C). Although the HR remained higher throughout life in TG^AC8^ vs WT (Fig. 8D), in aged TG^AC8^, the EF started to decline (Fig. 8E) between 14 and 19 months of age, whereas the left ventricular (LV) mass (Fig. 8F), the end diastolic volume (EDV) (Fig. 8G) and the end systolic volume (ESV) (Fig. 8H) increased, demonstrating a reduced heart performance, vs old WT. Interestingly, some hearts within the aged TG^AC8^ group were more dilated and had a greater reduction in EF than others (Fig. 8B, C). This heterogeneity in LV’s reduced function matches the increased heterogeneity present in aggregates (LC3^+^-puncta) and oxidized-lipoprotein accumulation (LF) in old TG^AC8^ vs old WT (Figs. 3Q and R and 7B–F, G, and I).

## Discussion

It is widely recognized that marked chronic catecholamine-induced cardiac stress leads to HF [21, 68], and that current treatments for CHF (β-adrenoreceptor blockers [69] and RAS inhibitors [70]), although effective, are suboptimal in amelioration of HF progression. Our TG^AC8^ heart model, in which cardiac-specific (α-MHC) AC8 over-expression markedly enhances AC activity and cAMP signaling, may be ideal for elucidating all the adaptations that must become engaged in response to incessant chronic activation of cardiac AC/cAMP/PKA/Ca^2+^ axis.

Our previous work in TG^AC8^ mice had shown that, up to 3–4 months of age [13], sustained upregulation of the AC/cAMP/PKA/Ca^2+^ axis activates numerous concentric adaptation mechanisms that protect heart health and function. Among other mechanisms, PQC, including the UPS and the autophagic machinery (Supplemental Tables 2–4), became more activated in TG^AC8^ to ensure more efficient proteostasis. The present study evaluated proteostasis in the TG^AC8^ heart in more detail, focusing on key players involved in the autophagic process at 3–4 months of age, and probed whether PQC mechanisms remained adequate in advanced age.

### Autophagy in the TG^AC8 ^heart at 3–4 months of age

Our results show that efficient modulation of LC3’s isotype-specific functions [22] and of the protein-adapter p62 for cargo recruitment [23] and selection [71] guarantees a more favorable autophagic process and effective clearance of damaged proteins, which are essential PQC features in the context of a high cardiac workload and energy demands. One manifestation of upregulated autophagy in TG^AC8^ vs WT is that LC3, and specifically its isoforms LC3A and LC3B, exhibits distinct expression patterns (Fig. 1A, B). Specifically in the young TG^AC8^ heart, LC3A orchestrates basal autophagy, and LC3A-mediated autophagy modulates the PQC network [72] to effectively resolve both aggresome formation and disposal of failing mitochondria by mitophagy [35] (together with FKBP8). We speculate that LC3A’s upregulation is important for efficient PQC in the “stressed-out” TG^AC8^ heart and that downregulation of LC3B is consistent with the idea that LC3A and LC3B are involved in different signaling pathways [35, 51, 72–74]. LC3 post-translational modifications (PTMs) by phosphorylation, that affect its activity [24, 25], were also differentially modulated in the young TG^AC8^ vs WT heart in a way that favored both early- and late-stages of autophagy: specifically, phosphorylation of LC3 at S12 by PKA/PKC [75], which inhibits autophagy by preventing autophagosome wall elongation, was significantly reduced, whereas its phosphorylation at T50 by STK3/4 [25], which promotes autophagolysosome fusion, was significantly increased.

Another manifestation of fine-tuned, regulated, efficient autophagy in TG^AC8^ vs WT was that the essential autophagic adaptor-protein [76] p62 and its phospho-forms p62^S405^ and p62^S349^ (and their ratios to total p62) were all significantly upregulated in TG^AC8^ vs WT at 3–4 months of age (Fig. 1E–I). p62 is an important key mediator of the crosstalk between UPS and autophagy [77] and facilitates the recruitment of components of the UPS machinery forming “gel-like” droplets (condensates) in response to various stressors [78]; additionally, it stabilizes aggresome formation [79] and clearance [80] by acting as a scaffold, thus improving the efficiency of degradation while, itself, becoming an autophagy substrate [81]. Moreover, p62^S405^ correlates to ubiquitinated cargo [27] (including protein aggregates [37]), cellular inclusion bodies (the aggregates of aggregates [42]), and polyubiquitinated mitochondria [27], whereas p62^S349^ has higher affinity for the *ubiquitin ligase adaptor Kelch-like ECH-associated protein 1* (KEAP1) [82], resulting in constitutive activation of the transcription factor *NF-E2-related factor 2* (NRF2) and regulation of its downstream signaling pathways [83] (with a positive feedback on p62 transcriptional activation itself [29]). Although p62 accumulation is normally linked to a scenario compatible with decreased autophagy/autophagy flux, within the context of the TG^AC8^ heart, we interpret the *transient* accumulation of p62 as an indication of higher induction of autophagy [84], of increased detection of ubiquitinated proteins in p62 clusters [28, 37] and of an upregulated antioxidative stress response (increased protein levels of Nrf2 [13] and higher sequestration of Keap1 within the droplets [85]). Thus, at 3–4 months, both PQC degradation mechanisms (UPS [13] and autophagy) are enhanced to ensure TG^AC8^ heart health and function, being this “working harder” an adaptation response to the sequelae evoked by the sustained activation of the AC/cAMP/PKA/Ca^2+^ axis.

Indeed, the number of autophagic events (Fig. 3C–G), following CQ treatment, was increased in young TG^**AC8**^, and endogenous LC3^+^-puncta counts, which correlate with autophagosomes, were *more numerous* although *smaller* (Fig. 3L, M), vs WT. Since cargo detection is regulated by flexibility in autophagosome size [86], *higher counts of autophagic events* in the context of *smaller* LC3^+^-puncta indicate increased *frequency of autophagosome formation* and enhanced “selective” autophagy in young TG^AC8^ vs WT. In addition, *smaller* (Fig. 6M) and *rounder* (Fig. 6L) cross-sectional areas within the mitochondrial network in young TG^AC8^ vs WT, together with a trend toward smaller values in their volumetric density, point to *accelerated mitophagy/autophagic flux* [87, 88] (increased physiological mitochondria fragmentation occurs just before the onset of mitophagy [89]).

However, remarkably, LC3II protein levels (Fig. 2A, B) did not accumulate in TG^AC8^ following CQ, and, in addition, LC3AI and LC3BI levels were *always decreased* (Fig. 2A, B), vs WT, whether LC3 was transcriptionally *upregulated* (LC3A) or *downregulated* (LC3B). We interpret this reduction in LC3I protein levels in the young TG^AC8^ heart to be the result of an *accelerated LC3 turnover* that prevents accumulation of LC3II at the lysosome, because of a *faster LC3I to LC3II processing* (increased levels of ATG4B (Supplemental Figs. 3, 4A) and ATG16L1 (Fig. 2D)), and a *faster degradation of LC3II* (increased levels of ACP2 (Supplemental Fig.  1C), and of several cathepsins [13], together with the upregulation of cathepsin L1 activity (Supplemental Fig. 1A-B)), compared to young WT. Additionally, it cannot be excluded that the lack of accumulation of LC3II and p62 in TG^AC8^, following CQ, is due to a faster clearance of CQ from the TG^AC8^ high-performing heart and to an enhanced autophagic flux in general. Our autophagic flux assessment conditions were optimized to detect LC3II accumulation (following CQ) in WT controls, and we reasoned the single CQ treatment of 3 h to be optimal for our transgene model, because it added minimal perturbation of Ca^2+^ levels (CQ has been shown to affect Ca^2+^ signaling both by suppressing Ca^2+^ channels in some cell types [90] and by increasing Ca^2+^ concentration in others [91]), yet allowing us to still see the effect before the drug was already cleared from the system (drug clearance in the heart occurs earlier compared to other organs [92]).

Thus, *in toto*, the present results, together with those of our previous study showing increased proteasome activity without accumulation of *insoluble* aggregates, and increased levels of proteins involved in the autophagic machinery [13], strongly suggest that UPS and autophagic pathways concurrently operate in a more efficient mode in the young TG^AC8^ heart to manage its state of chronic, marked cardiac cAMP-derived stress [46, 93].

### PQC mechanisms in aged TG^AC8^and WT

#### Autophagy/mitophagy

Autophagy markers LC3 (Fig. 3A) and ATG4B (Supplemental Figs. 3 lines 13–24, 7) became significantly downregulated with age in both TG^AC8^ and WT (WB were under-powered (*N* = 3) due to the constraint of loading groups on the same membrane to be comparable). In addition, the number of early autophagic figures (Fig. 3C, D, and G) and the number/size of autophagosomes (LC3^+^-puncta) (by TEM quantification and fluorescence microscopy IHC) were also decreased (Fig. 3L, M), whereas the number of late autophagic events (Fig. 3E–G) and the number of lysosomes/late endosomes (LAMP1^+^-puncta) (Fig. 3I, J) dramatically increased.

Although the present results clearly point to downregulation of induction of autophagy and enhanced clearance impairment, i.e., less efficient autophagy, due to aging, per se, several pieces of evidence suggest that some PQC mechanisms fail more severely than others in a context of chronic exposure (up to 21 months) to marked cardiac cAMP-induced stress and that autophagy is more severely compromised in aged TG^AC8^ than in age-matched WT hearts.

Firstly, the nuclear to cytoplasm ratio of TFEB (by WB) was significantly reduced in old TG^AC8^ (Fig. 4G–J), in a context of upregulation of several 14–3-3 s transcripts, vs old WT (Fig. 4K–Q). This reduction clearly demonstrates that the CLEAR network and the autophagic program are more severely suppressed in the aged TG^AC8^ than that due to “normal” aging in WT [38, 39]. Secondly, LC3A was transcriptionally upregulated [13] and its carrier-flux accelerated in aged TG^AC8^ (Fig. 4A) but *overall* autophagic flux was reduced more than in WT (reduced protein levels of p62, ATG16L1, ALIX, and ATG4B, changes that we were still able to assess although we had the limitation of a small *N* (*N* = 5)), following CQ treatment (Fig. 4C–E and Supplemental Figs. 3, 4A, respectively). Activation of LC3A has been linked specifically to inhibition of proteasome activity as a general stress response [72]; additionally, in conditions of altered autophagy, LC3A-silencing (by promoter methylation) has been shown to prime cells for aggresome formation to achieve cellular homeostasis [94]. Indeed, the fact that endogenous LAMP1^+^- and LC3^+^-puncta covered a smaller % of cell area in TG^AC8^ vs WT of advanced age (Fig. 3K, N) and were highly heterogeneous in size (Fig. 3O, P) portrays a scenario compatible with increased aggresomes [42, 72], the “aggregate of aggregates” that cannot be removed from the cytosol by the UPS [95], because they are covalently cross-linked and encaged by intermediate filament proteins [96]. Hence, we correlate enhanced LC3A levels and the accelerated LC3A-flux in aged TG^AC8^ to the presence of increased protein aggregates that lead to a more severe aging process, per se, in TG^AC8^ vs WT, a compensation mechanism that can be maladaptive in disease conditions that contribute to the remodeling of the myocardium under stress [94]. Increased LC3A-flux in aged TG^AC8^ vs WT is another indication of oxidized-lipoprotein accumulation in the context of chronic upregulation of the AC/cAMP/PKA/Ca^2+^ axis in old TG^AC8^.

Because LAMP1 is also a marker of late endosomes, increased numbers of endogenous LAMP1^+^-puncta (Fig. 3I) vs LC3^+^-puncta suggest that cargo detection and transport differ in aged TG^AC8^ vs WT. In addition, accumulation of LF bodies (Fig. 7A–F) and of insoluble p62^+^-inclusions (Fig. 7L, M) in aged TG^AC8^, together with the presence of black-to-blueish pigments (Fig. 7G) and of LC3^+^-aggregates highly heterogeneous in size in CMs (Fig. 7H, I), indicate greater lysosomal impairment [97] and differences in cargo accumulation in the old TG^AC8^ vs WT heart. The buildup of numerous small pre-aggresomal bodies resulting from compartmentalizing as a cytoprotective mechanism [96, 98] at a younger age further contributes to increased aggregate accumulation in aged TG^AC8^ vs WT during late stages of autophagy.

Thus, the optimized, highly efficient autophagic flux that accommodated proper cargo clearance in young TG^AC8^ becomes dysfunctional as TG^AC8^ ages and would clearly be insufficient for proper clearance of aggregates/inclusions of aberrant size, compared to the young TG^AC8^ and the aged WT.

The mitochondrial network was more severely disrupted in old TG^AC8^ (Fig. 6H, I), vs WT. Specifically, *individual cross-sections per area* within the mitochondrial network were increased in aged TG^AC8^ vs WT, in a context of *reduced circularity and an increment in volumetric density* (Fig. 6M, P). This scenario portrays a less interconnected mitochondrial network in aged TG^AC8^ vs WT, and, together with the significant increase in the ratio of S-OPA1 to L-OPA1 (Fig. 6N, O), points to a more severe decline in mitochondrial fitness in the old TG^AC8^ heart, vs age-matched WT. Significant alterations in mitochondrial morphology, reflected by mitochondrial dysfunction and increased reactive oxygen species (ROS) production, in response to LC3A-induced autophagy, have been shown to result in altered mitochondrial dynamics and stress-induced senescence [72]. Hence, specifically in the in old TG^AC8^ heart, excessive stress-induced processing of OPA1 by OMA1 [52], in the context of sustained chronic cardiac stress triggers mitochondrial fragmentation accelerating mitochondrial fission [56], leading to disruption of the mitochondrial network and to increased LC3A-expression and accelerated LC3A-flux in old TG^AC8^ heart, vs age-matched WT. The higher cellular-stress response and the need of a “specialized autophagy” to maintain cellular/mitochondrial homeostasis further emphasize the intricate interplay between autophagy and mitochondrial dynamics [72].

Although protein levels of cargo receptors involved in *both* canonical (Fig. 6O–Q) and non-canonical (Fig. 6R) mitophagy signaling were significantly upregulated in aged TG^AC8^ vs WT, reduced signaling for disposal of oxidized-lipoproteins, together with imbalanced levels of OPA1, decreased protection from HSP60, and the greater mitochondrial fragmentation in old TG^AC8^, suggest that despite the *concurrent activation* of *both* canonical and non-canonical mitophagy signaling in old TG^AC8^, mitophagy failure is more severe, vs aged WT.

Taken together, persistent chronic cardiac cAMP-derived stress in the TG^AC8^ heart exacerbates the odds to the already increased cellular stress [58] due to aging, per se, and decreases protection of the mitochondrial matrix [49] and its associated functions [99] in the old TG^AC8^ heart vs age-matched WT.

Desmin ubiquitination and cleavage and desmin-PAOs accumulation (assessed by protein fractionation and WB) were all significantly increased in old TG^AC8^ (Fig. 7J, K), vs WT. Negative modulation of desmin’s physical properties and assembly, and of its PTMs, has been shown to affect mitochondrial positioning [63, 100], therefore compromising mitochondrial function, and to lead to the build-up of cardiac PAOs. Hence, the cardiac contractile machinery likely becomes disrupted by desmin disorganization and increased desmin-PAOs more in aged TG^AC8^ than in old WT.

In addition, the number of cardiomyocytes expressing HSP27^S82^ (assessed by fluorescence microscopy IHC) was also elevated in aged TG^AC8^ vs WT, and this increased expression co-localized with p62^+^-inclusions (Fig. 7L, M). HSP27^S82^ expression at sarcomeres (and specifically cardiac troponin T) [67, 101] is upregulated in stress conditions (including cardiac injury [102]) enhancing heart tolerance to stress [103]. Upregulation of HSP27^S82^ indicates increased heart damage in aged TG^AC8^ and points to the presence of damaged HSP27^S82+^-CMs (with a thinner LV wall and a sclerotic stroma) exactly at the site of “garbage” accumulation (p62^+^-aggregates), compared to old WT.

Therefore, failure of PQC mechanisms leads to poor cardiomyocyte “health,” a phenotype consistently associated with the context of cardiomyopathy and eventual HF [7]. Indeed, myocardial function and cardiac structure progressively declined from 6 to 21 months of age in aged TG^AC8^ leading to a failing hypertrophic and dilated heart at 19 months of age, a sign of *cardiac aging*.

#### Proteasome

Although proteasome activity did not differ between genotypes at older ages, between 3–4 and 18 months, both proteasome function (Fig. 5A) and protein translation rates (Fig. 5E) progressively declined in TG^AC8^ but not in WT. Although a reduced protein synthesis is in accordance with a “normal” aging process [45, 104], cardiac proteasomal insufficiency has been linked to accumulation of aggregates [96]. Accumulation of *insoluble* aggregates was increased in aged TG^AC8^ vs WT, whereas *soluble* aggregates (soluble proteins undergoing normal turnover and misfolded proteins *en route* to degradation by the UPS) became significantly reduced in *both* genotypes at 18 months (Fig. 5B, C). Since protein insolubility increases with age, per se, and misfolded proteins are more “aggregate-prone” [42], with aging, we interpret the greater accumulation of *insoluble misfolded proteins* in aged TG^AC8^ vs old WT, to indicate that the proteasome of the old TG^AC8^ heart is “out-won” in the competition for misfolded substrates prior to their aggregation [105]. However, the reduction in protein synthesis in TG^AC8^ with aging suggests that, exactly as in the WT, the TG^AC8^ heart optimizes energy use, as age advances, by reducing the formation of de novo proteins in order to focus on repairing the existing proteome, by directing chaperons activity toward repair of existing proteins rather than to the folding of new ones [45, 104, 106].

In conclusion, in response to chronic cardiac AC-dependent cAMP-stress, *protective concentric signaling circuitry becomes activated*, manifested as increased autophagy/autophagic flux and proteasome activity, and *maintenance of PQC preserves heart health* in this context of markedly sustained chronic cardiac stress *early in life*. However, proteasomal insufficiency and a compromised PQC in the older TG^AC8^, in the context of a dysregulated autophagic flux, *accelerate cardiac aging*, leading to cardiac damage including desminopathy, severe loss of cardiac function, and cardiomyopathy.

### Future directions

Age-associated cardiovascular changes are currently largely under-appreciated, and aging, per se*,* in our opinion, is a disease that becomes the dominant risk factor for other clinical syndromes that have been, and continue to be, referred to as “cardiovascular diseases” [107]. Clearly, more work is needed to address how aging, per se, figures into failures that occur in the cardiovascular diseases, in order to define underlying disease mechanisms and to integrate and translate these discoveries into potential novel therapeutics.

Sex as a biological variable is also an under-appreciated aspect of biomedical research [108], and contemporary data have demonstrated differences, compared to males (humans/animals) in most, if not all, physiological systems, including the cardiovascular function [109, 110].

## Supplementary Information

Below is the link to the electronic supplementary material.

Supplementary file1 (PPTX 10.9 MB)

## Data Availability

All data supporting the findings of this study are available within the paper and its Supplementary Information.
