# Supplementary material for: Failure of efficient cardiac proteostatic adaptations to chronic cAMP-stress is associated with accelerated heart aging
Source: GeroScience. 2025 Sep 1;48(3):3557–89. doi: 10.1007/s11357-025-01851-y (PMC13356016; doi:10.1007/s11357-025-01851-y)
Supplement: Supplementary file 1 — Supplementary file1 (PPTX 10.9 MB) [file 11357_2025_1851_MOESM1_ESM.pptx]

## Slide 1
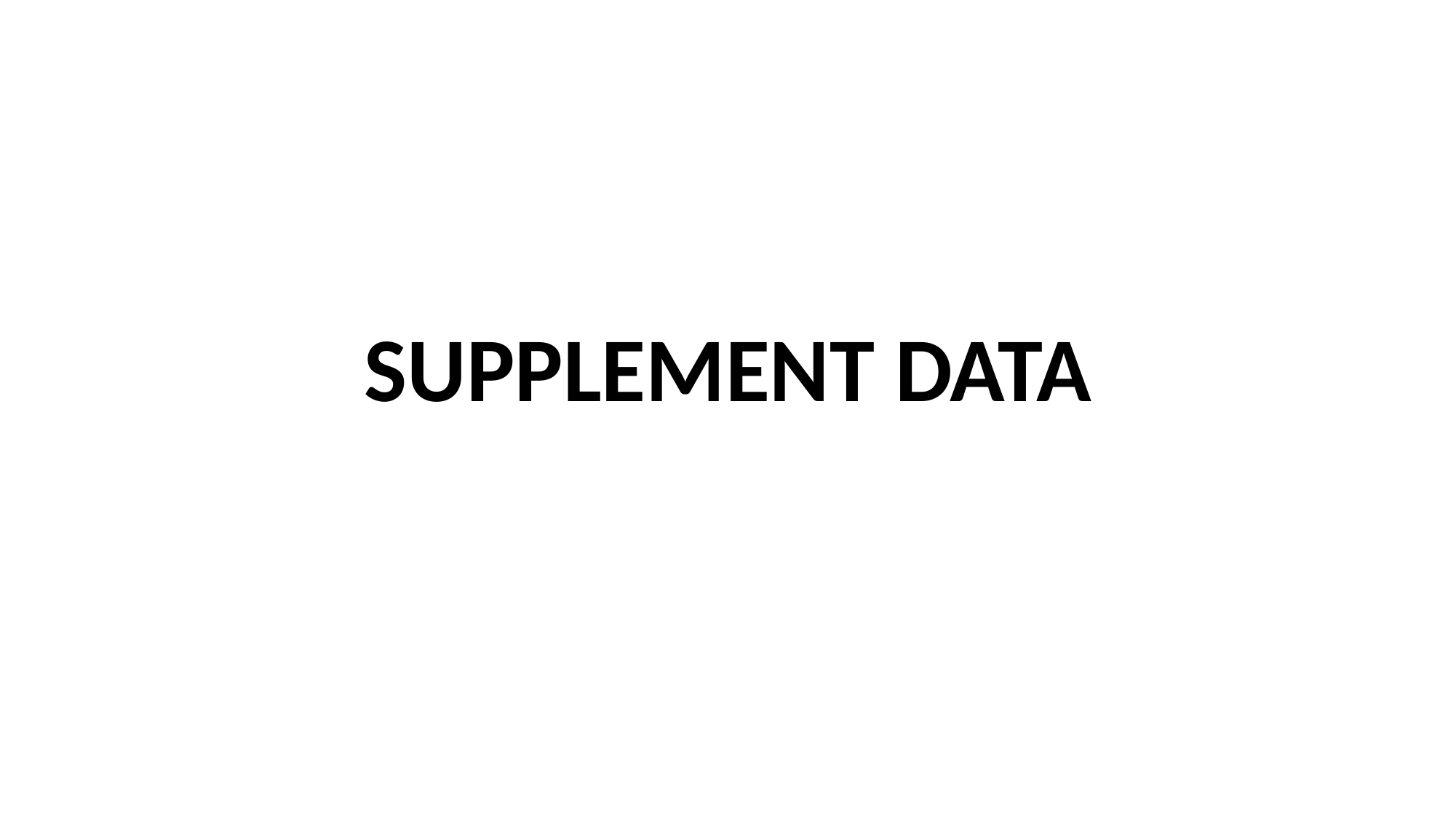

SUPPLEMENT DATA

## Slide 2
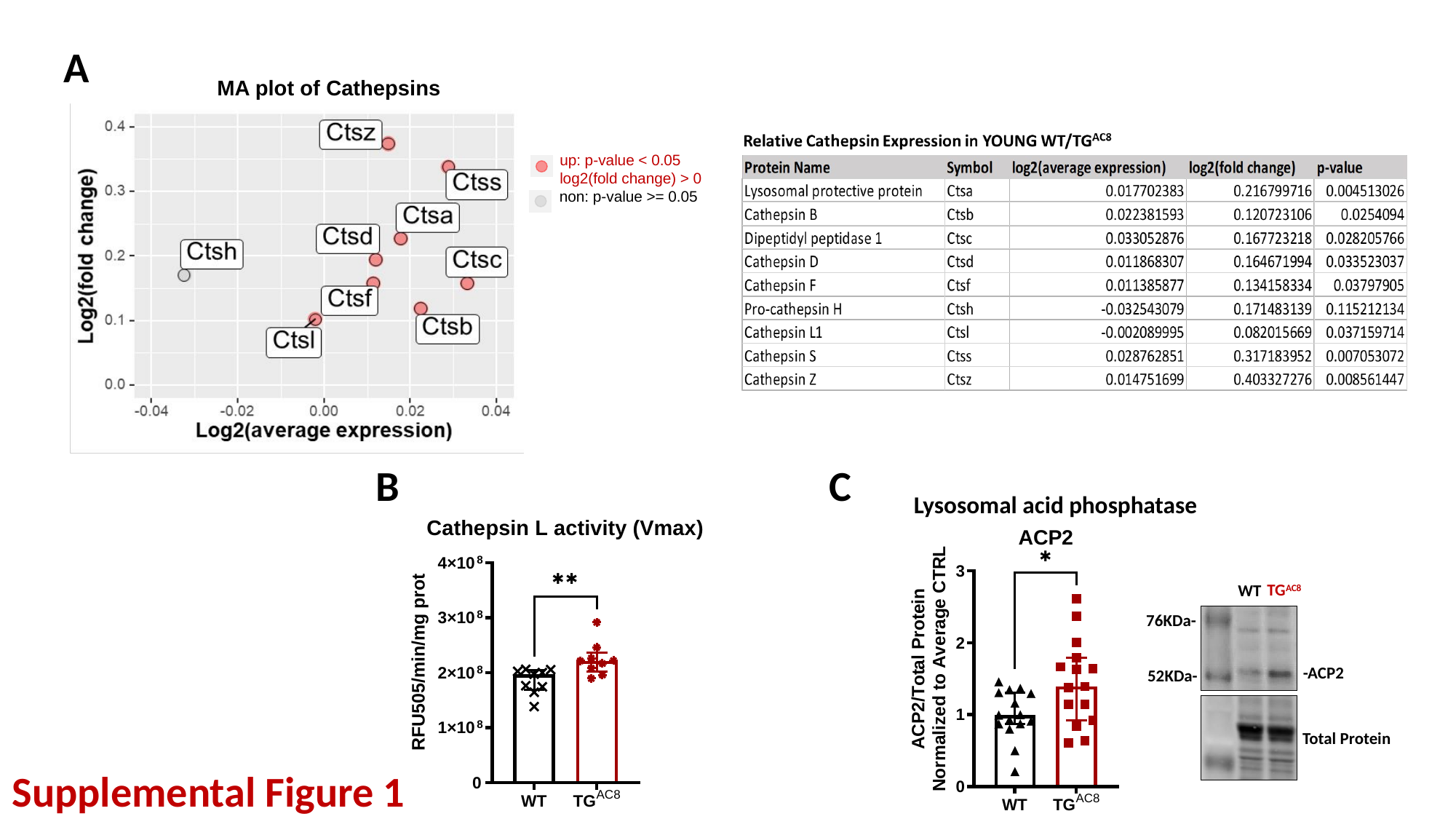

A
MA plot of Cathepsins
 up: p-value < 0.05
 log2(fold change) > 0
	 non: p-value >= 0.05
B
C
Lysosomal acid phosphatase
TGAC8
WT
76KDa-
-ACP2
52KDa-
Total Protein
Supplemental Figure 1

## Slide 3
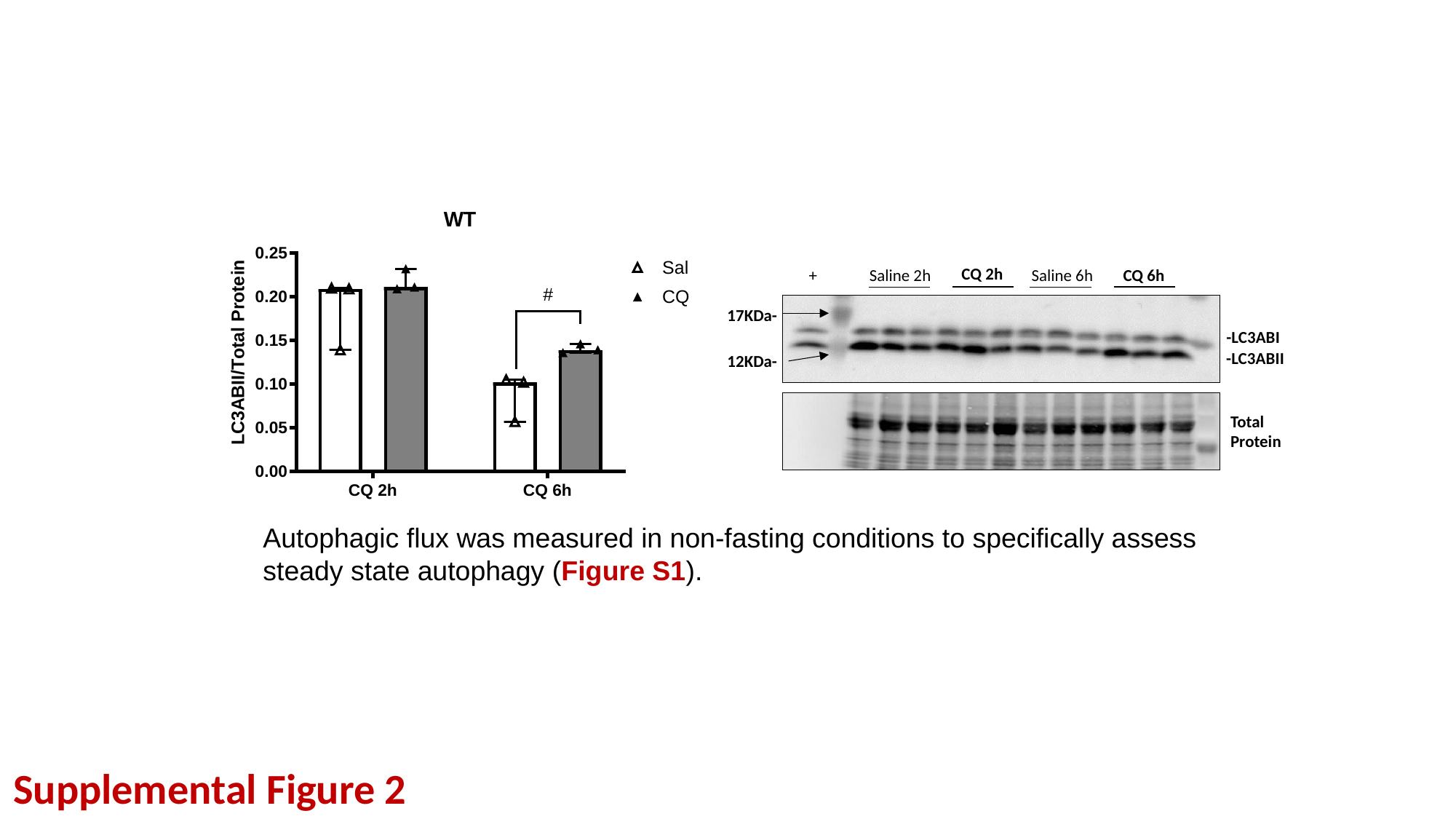

CQ 2h
Saline 2h
Saline 6h
CQ 6h
+
17KDa-
-LC3ABI
-LC3ABII
12KDa-
Total
Protein
Autophagic flux was measured in non-fasting conditions to specifically assess steady state autophagy (Figure S1).
Supplemental Figure 2

## Slide 4
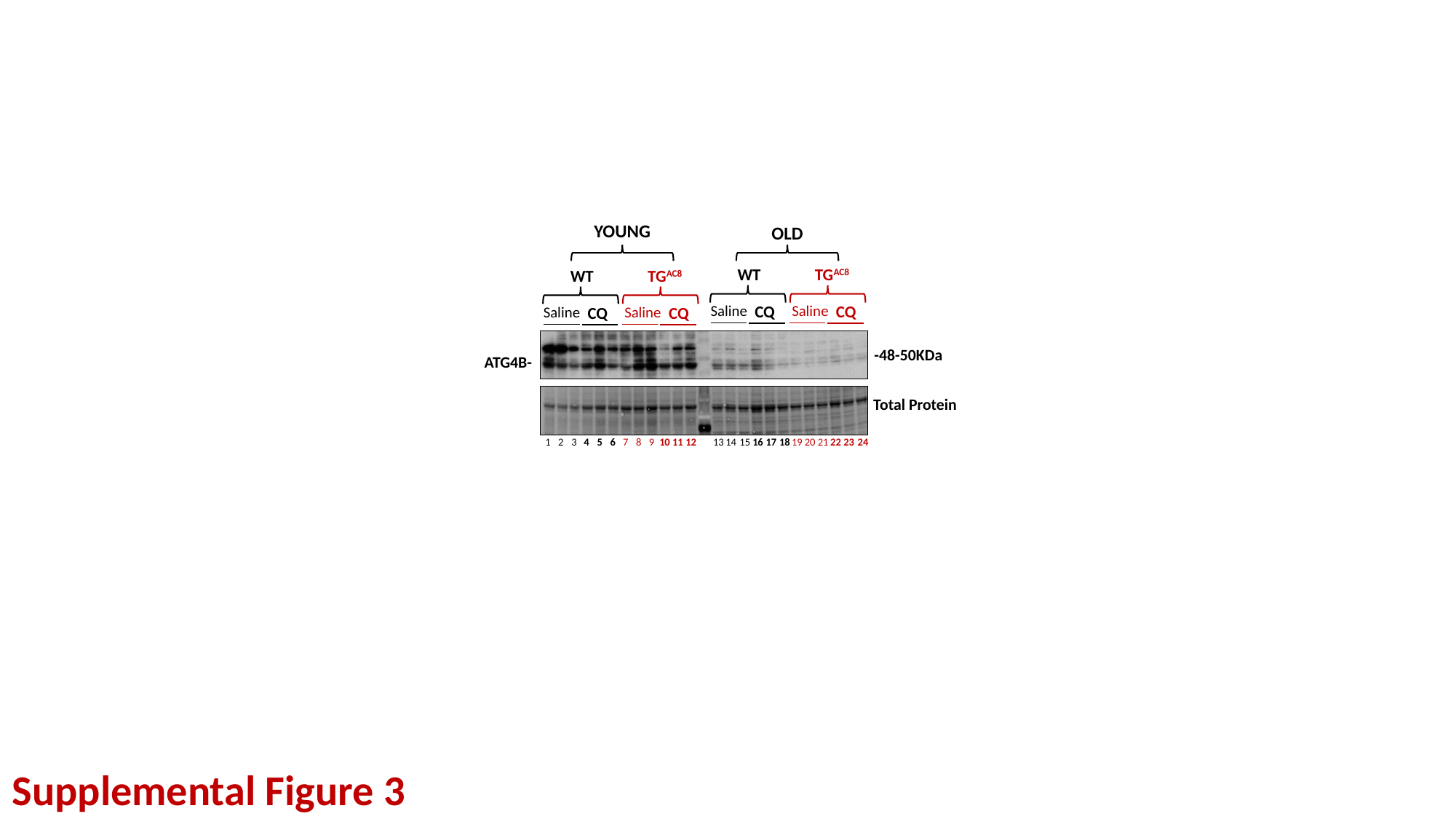

YOUNG
OLD
WT
TGAC8
Saline
CQ
Saline
CQ
WT
TGAC8
Saline
CQ
Saline
CQ
-48-50KDa
ATG4B-
Total Protein
20
22
19
1
2
3
4
5
6
7
8
9
10
11
12
13
14
15
16
17
18
21
23
24
Supplemental Figure 3

## Slide 5
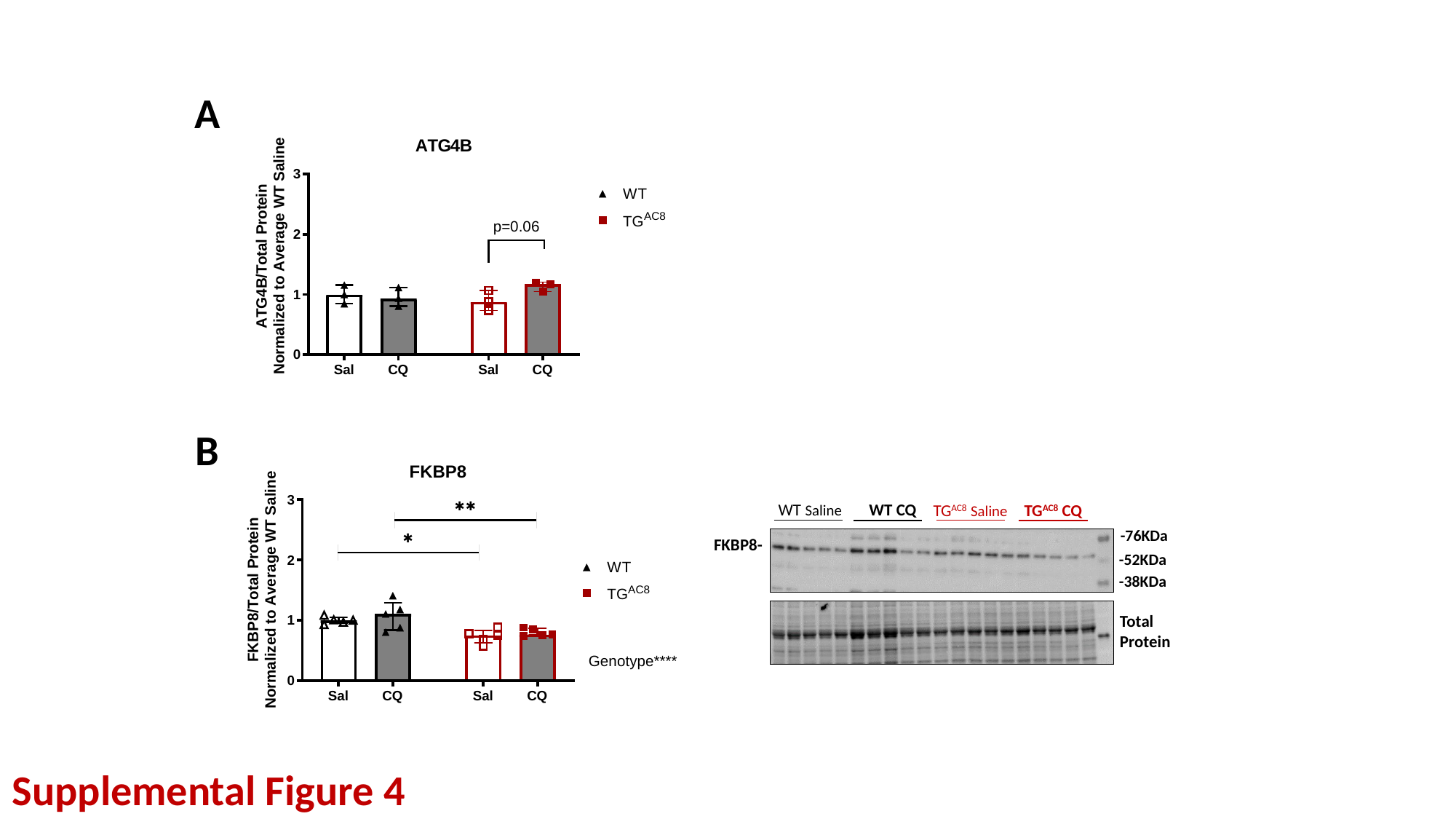

A
B
WT Saline
WT CQ
TGAC8 Saline
TGAC8 CQ
-76KDa
FKBP8-
-52KDa
-38KDa
Total
Protein
Supplemental Figure 4

## Slide 6
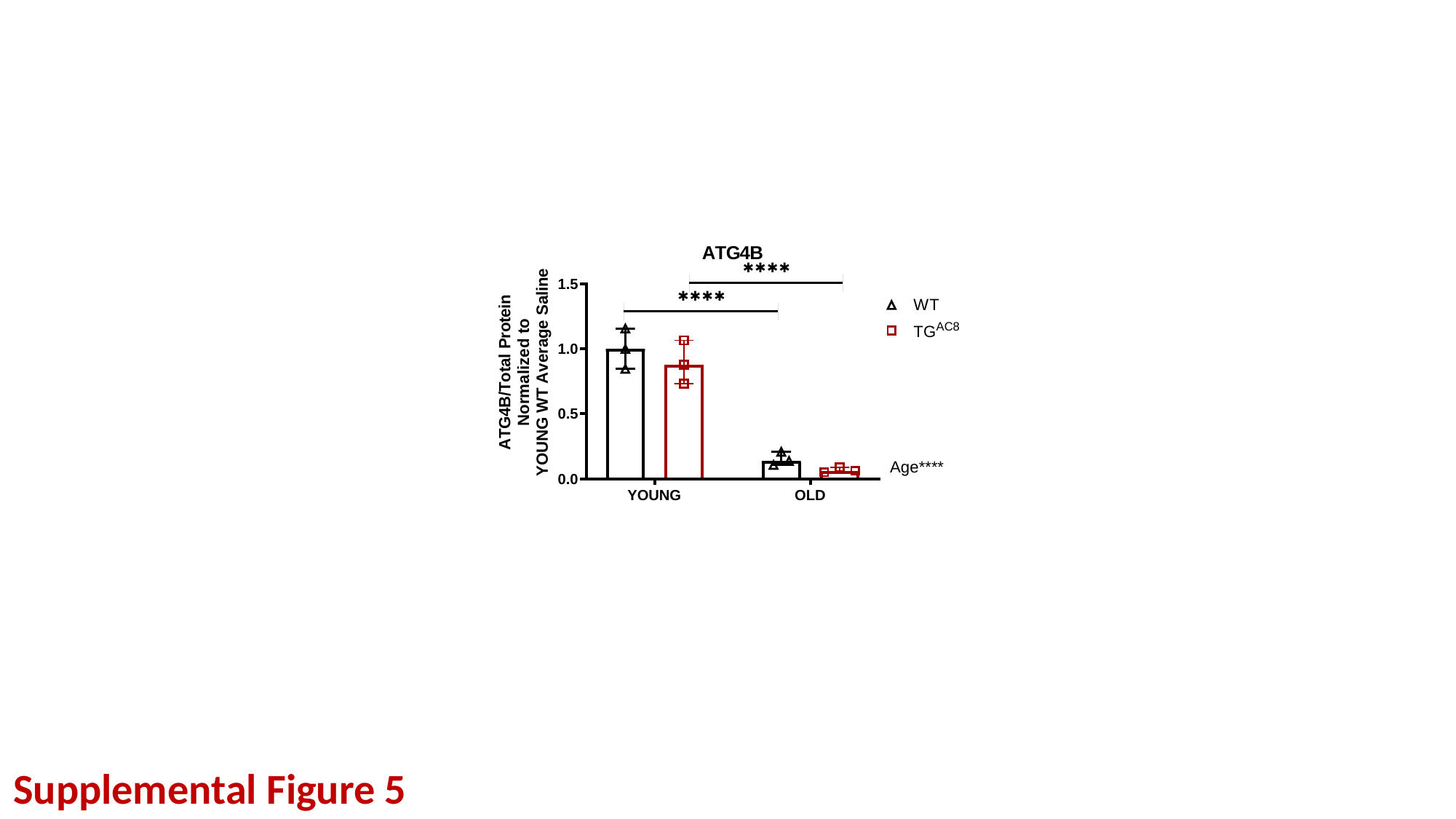

Supplemental Figure 5

## Slide 7
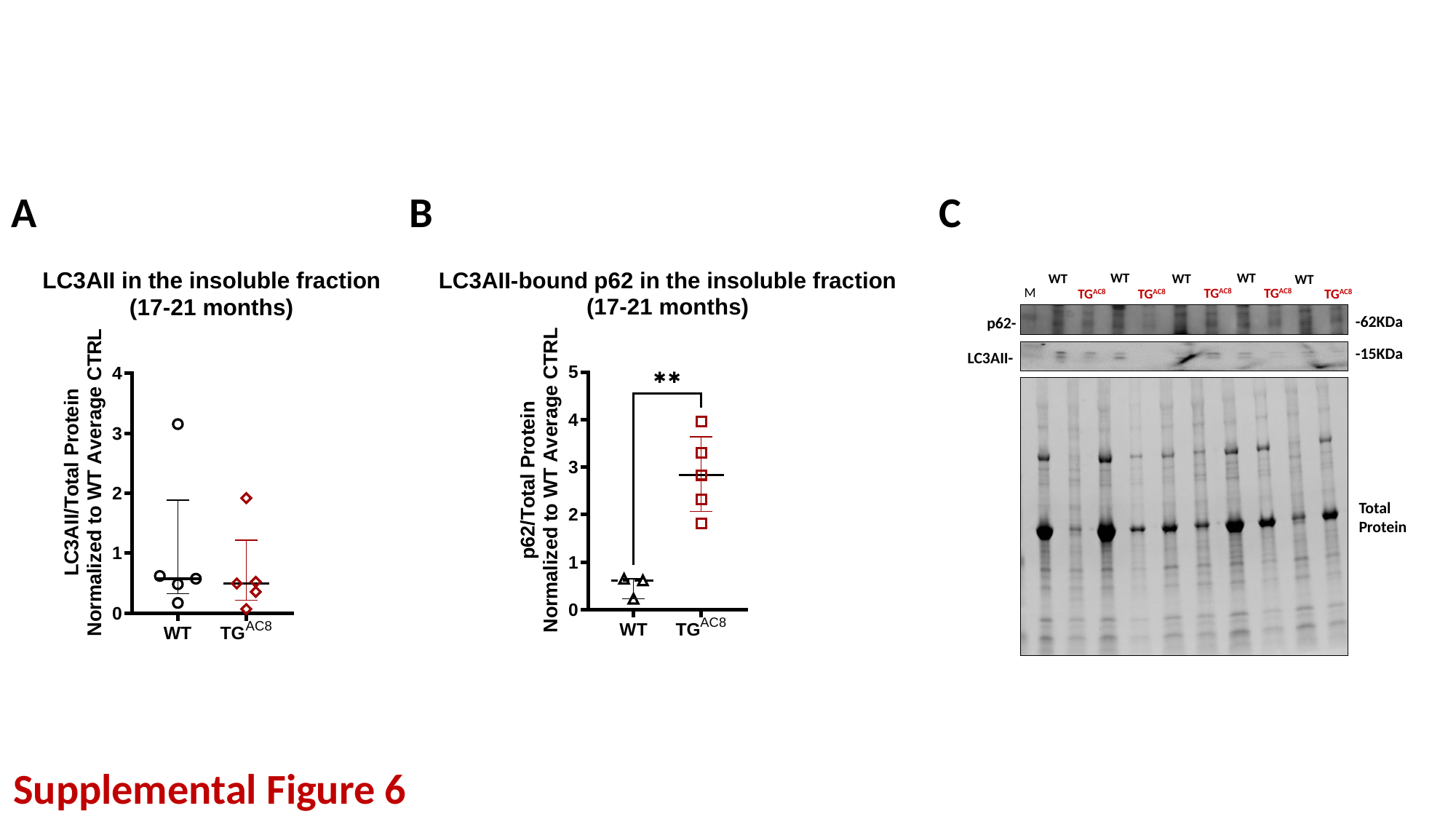

C
A
B
WT
WT
WT
WT
WT
M
TGAC8
TGAC8
TGAC8
TGAC8
TGAC8
-62KDa
p62-
-15KDa
LC3AII-
Total
Protein
Supplemental Figure 6

## Slide 8
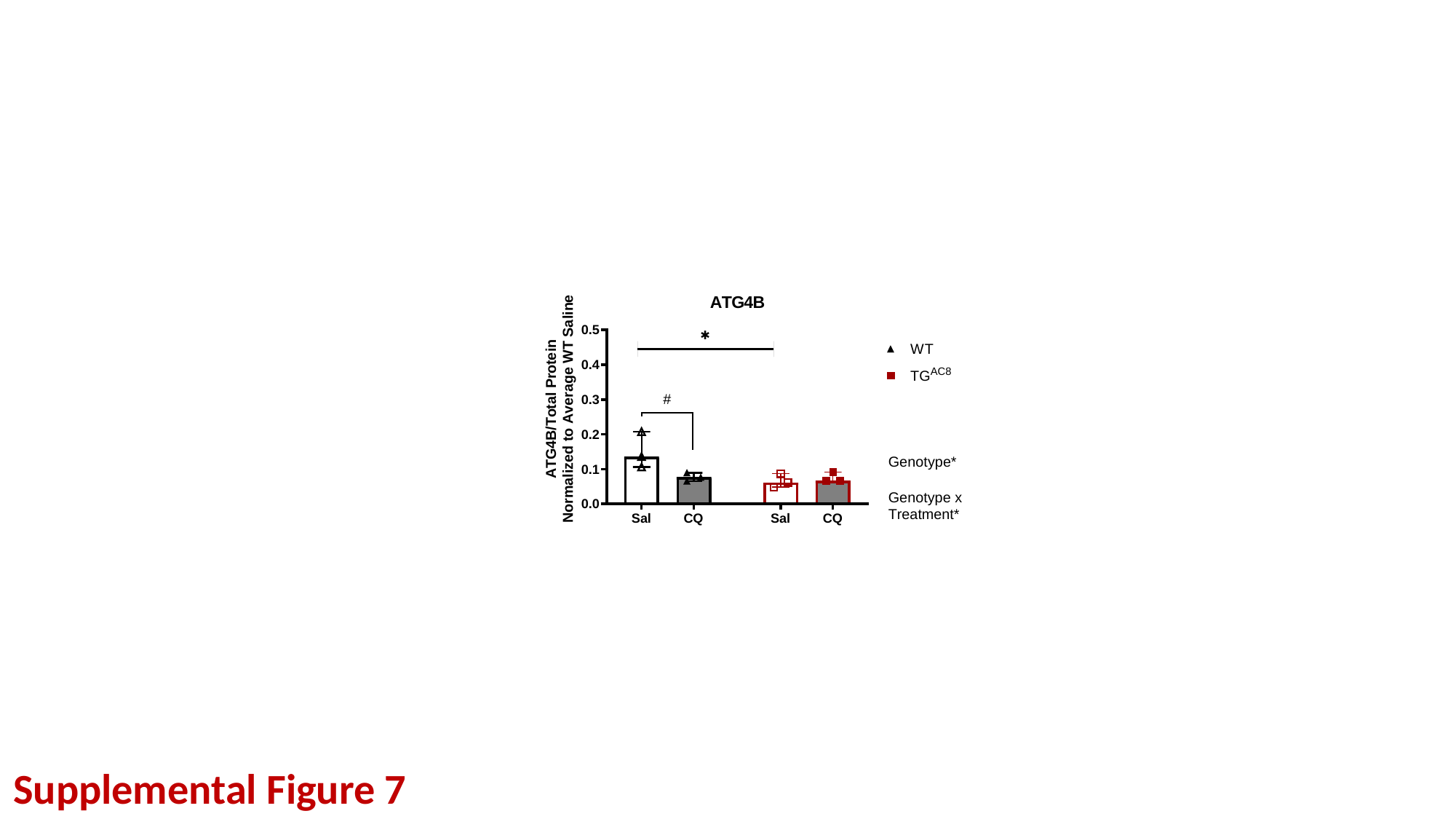

Supplemental Figure 7

## Slide 9
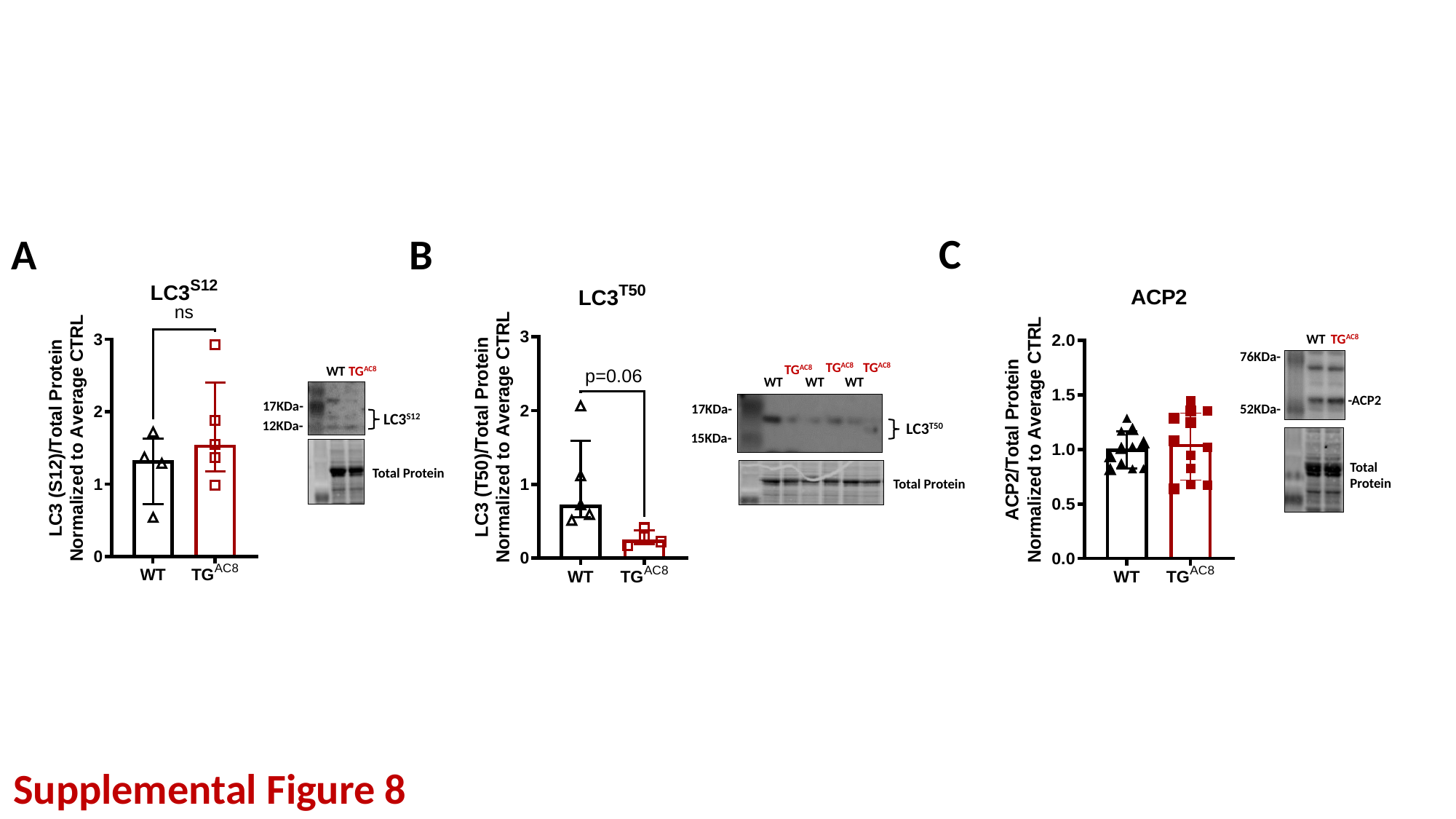

C
A
B
WT
TGAC8
17KDa-
LC3S12
Total Protein
12KDa-
TGAC8
TGAC8
TGAC8
WT
LC3T50
15KDa-
Total Protein
17KDa-
WT
WT
TGAC8
WT
76KDa-
-ACP2
Total Protein
52KDa-
Supplemental Figure 8

## Slide 10
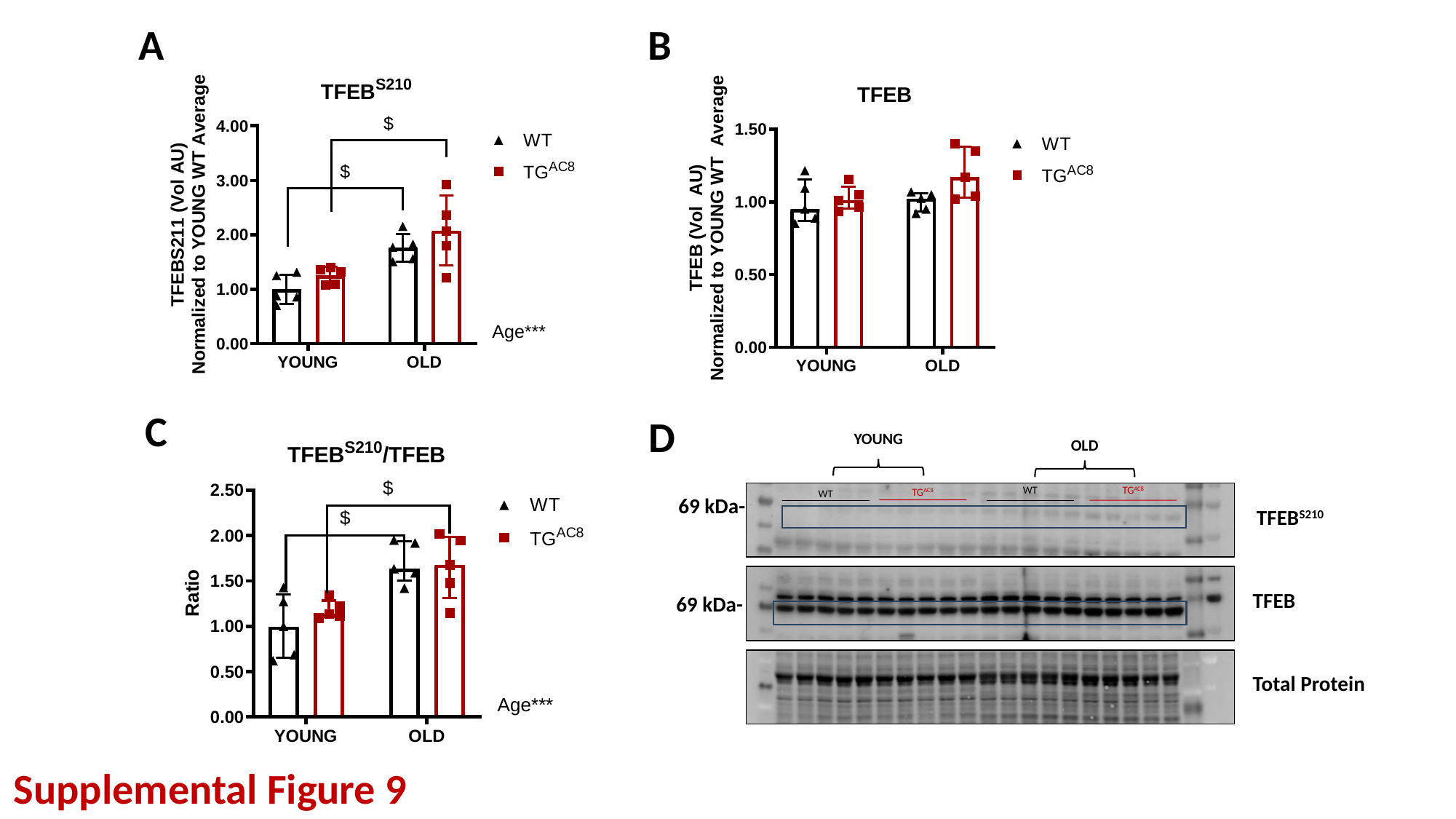

A
B
C
D
YOUNG
OLD
TGAC8
WT
TGAC8
WT
69 kDa-
TFEBS210
TFEB
69 kDa-
Total Protein
Supplemental Figure 9

## Slide 11
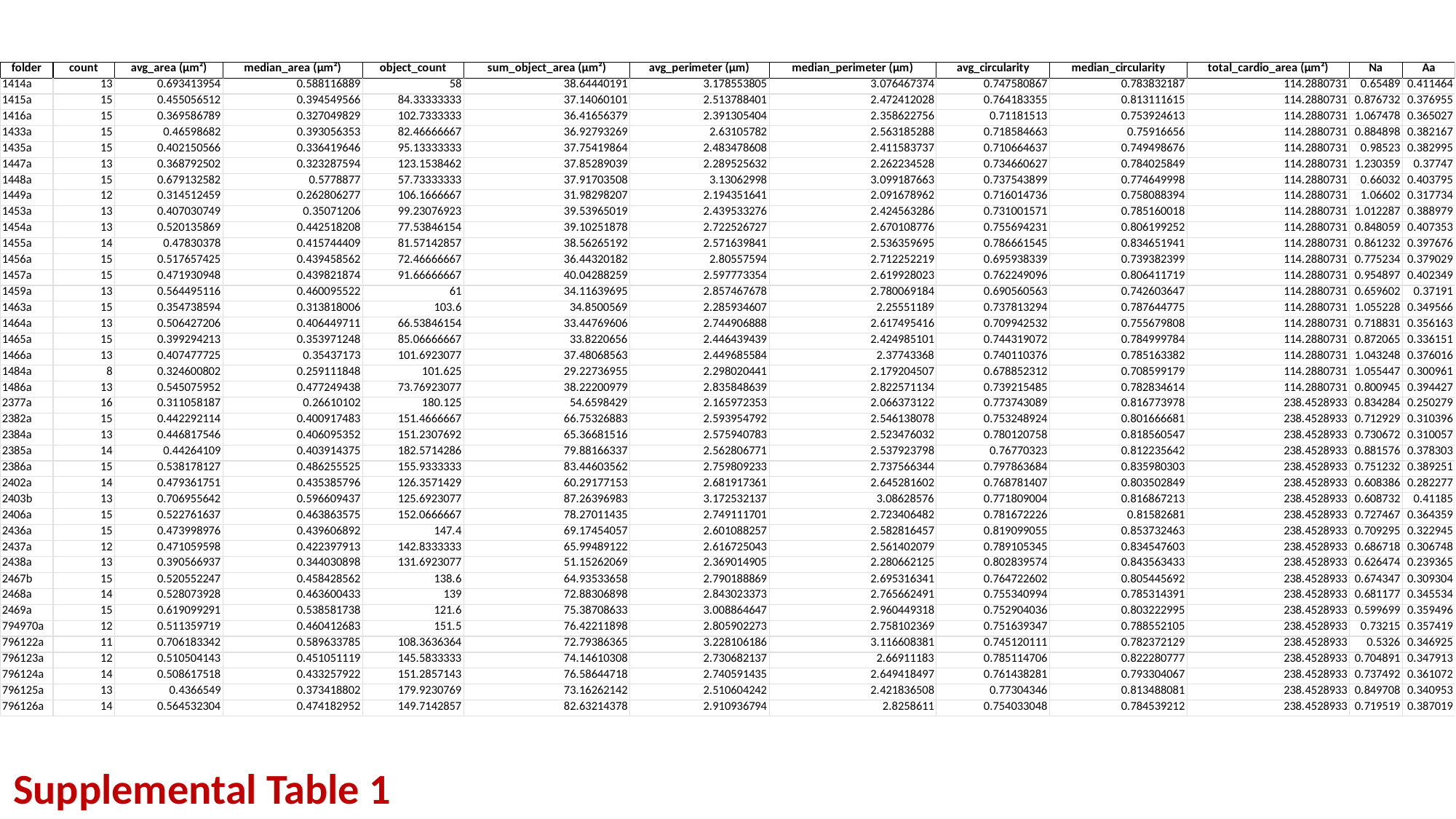

Supplemental Table 1

## Slide 12
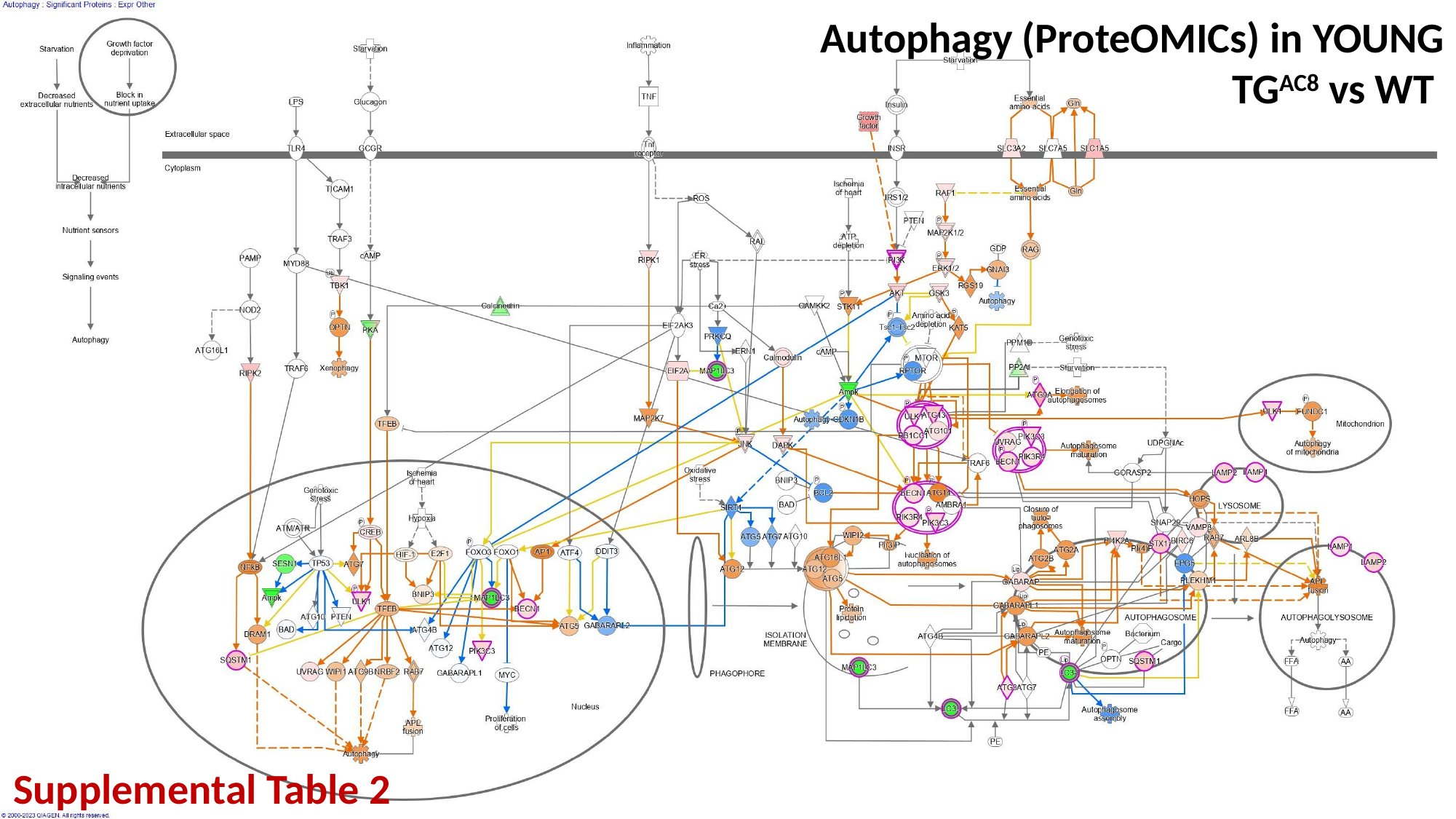

Autophagy (ProteOMICs) in YOUNG TGAC8 vs WT
Supplemental Table 2

## Slide 13
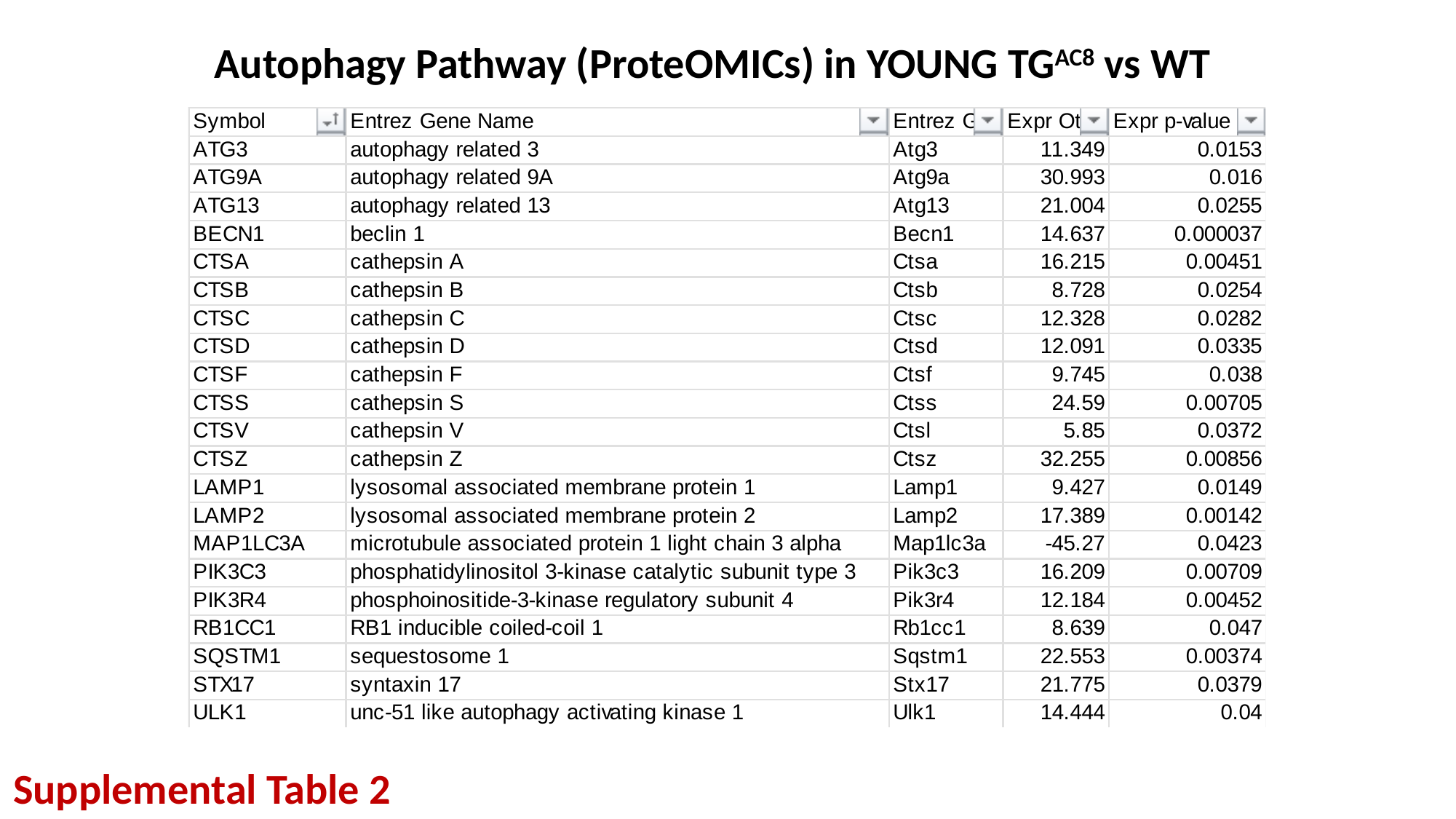

Autophagy Pathway (ProteOMICs) in YOUNG TGAC8 vs WT
Supplemental Table 2

## Slide 14
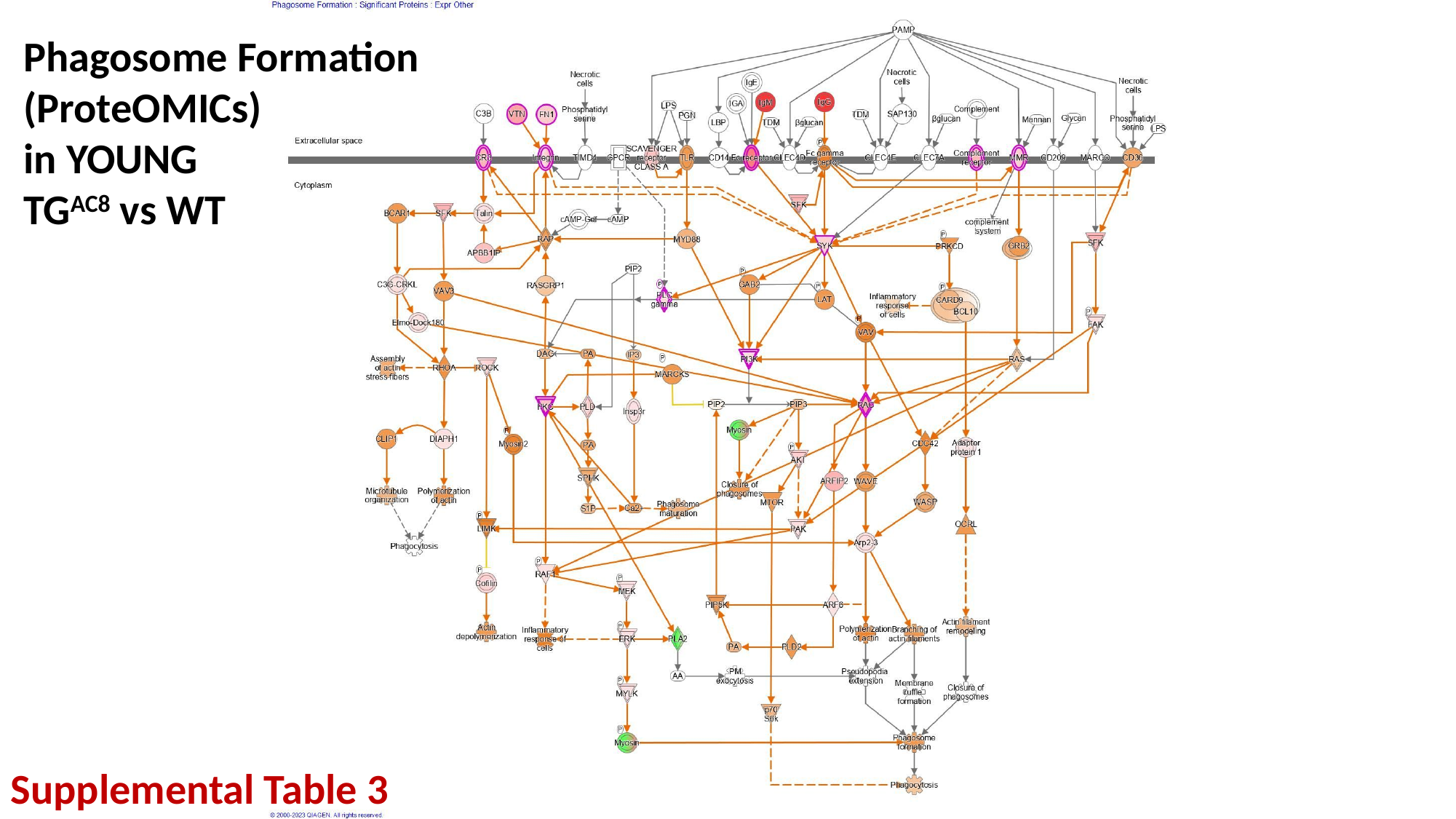

Phagosome Formation (ProteOMICs)
in YOUNG
TGAC8 vs WT
Supplemental Table 3

## Slide 15
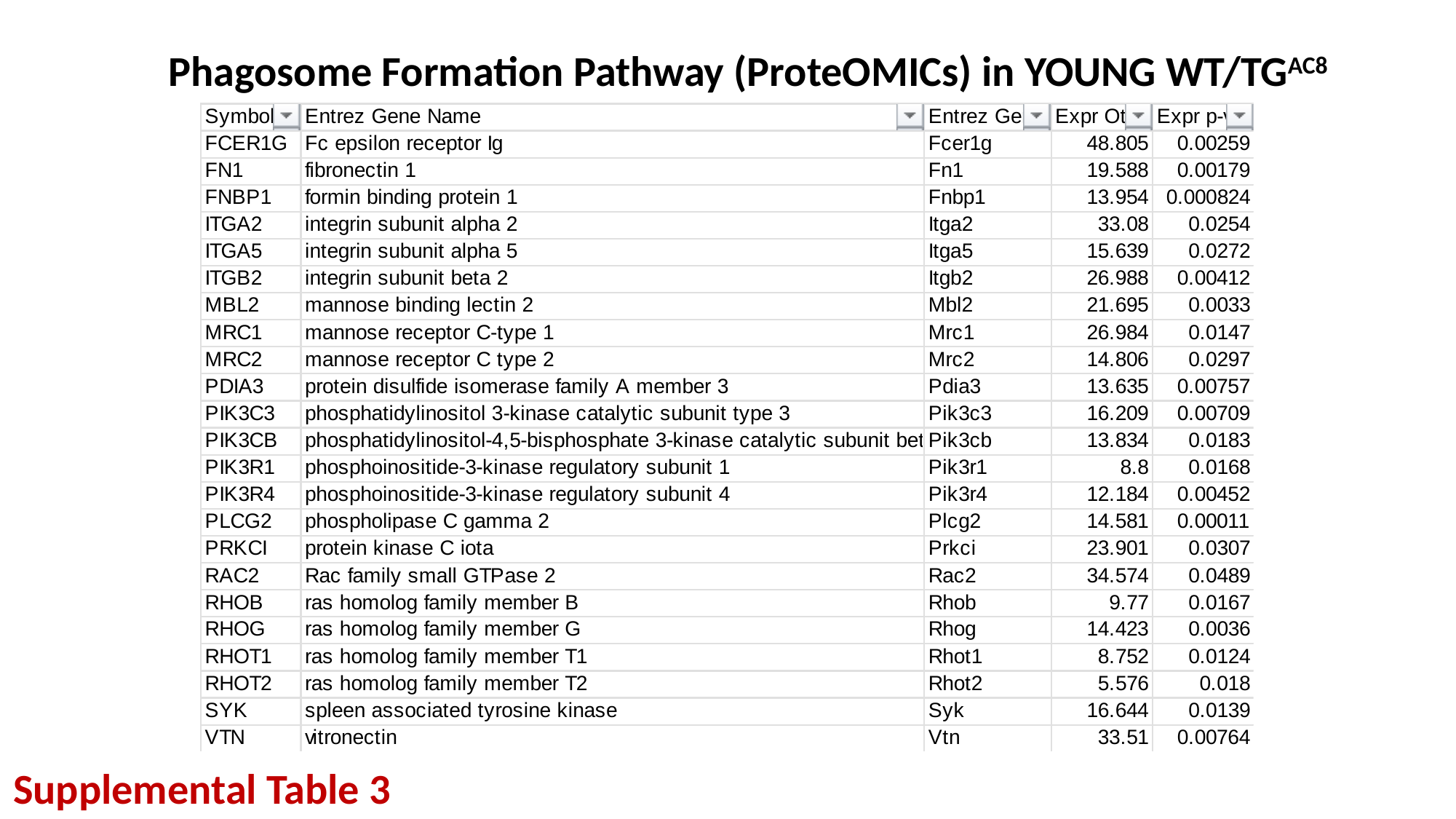

Phagosome Formation Pathway (ProteOMICs) in YOUNG WT/TGAC8
Supplemental Table 3

## Slide 16
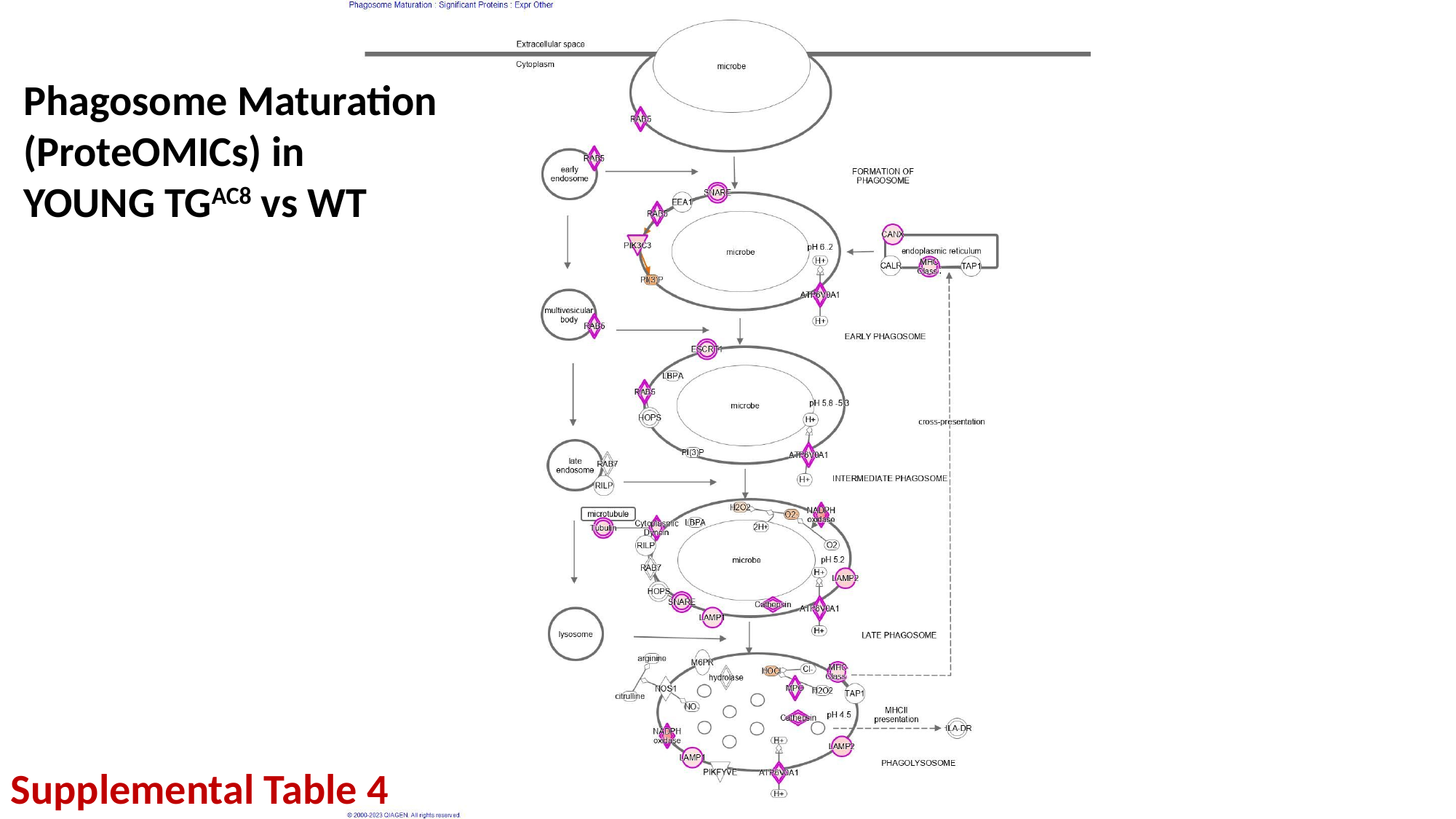

Phagosome Maturation (ProteOMICs) in YOUNG TGAC8 vs WT
Supplemental Table 4

## Slide 17
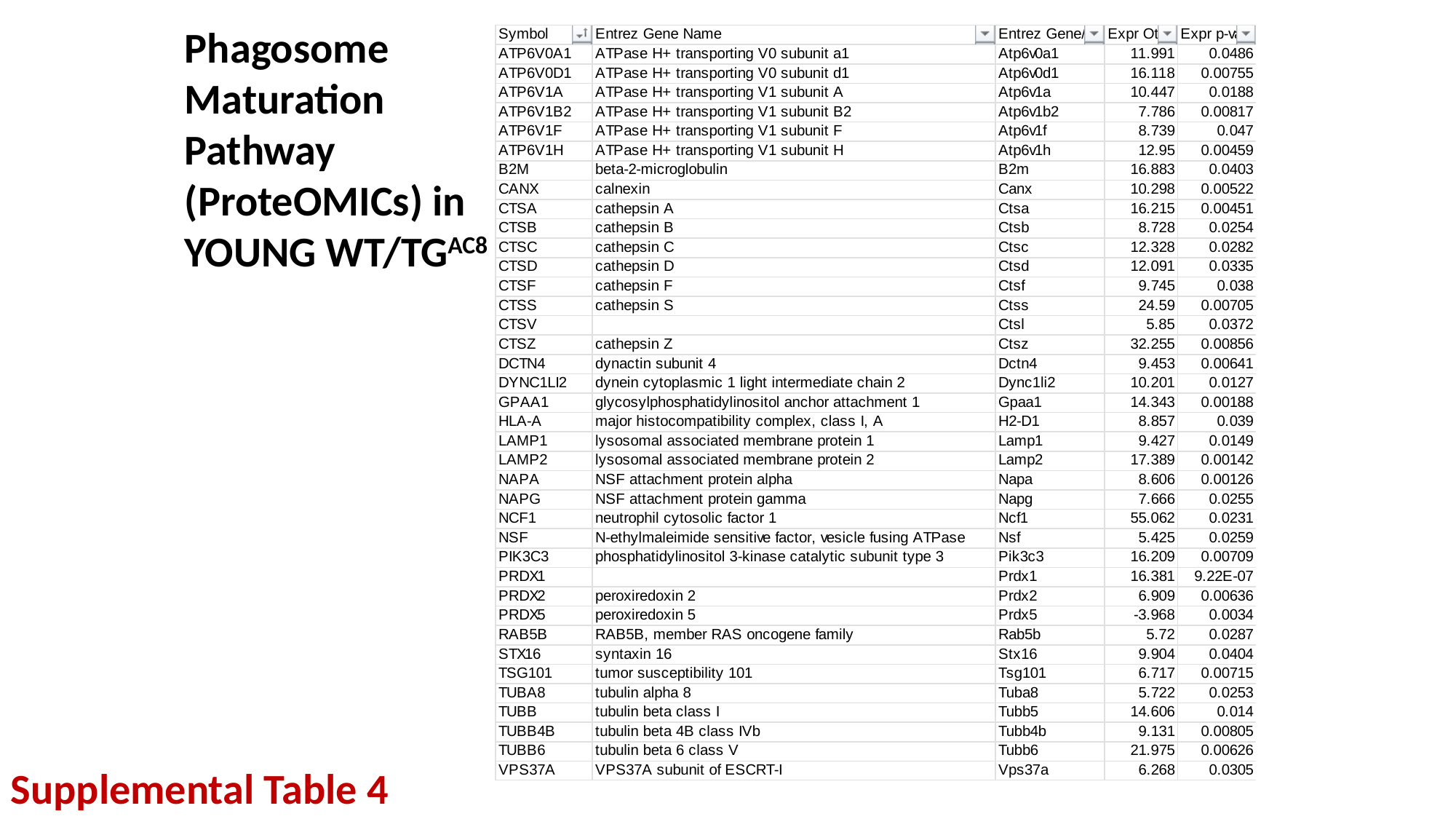

Phagosome Maturation
Pathway (ProteOMICs) in YOUNG WT/TGAC8
Supplemental Table 4
